# KMT2C/KMT2D-dependent H3K4me1 mediates changes in DNA replication timing and origin activity during a cell fate transition

**DOI:** 10.1016/j.celrep.2025.115272

**Published:** 2025-02-04

**Authors:** Deniz Gökbuget, Liana Goehring, Ryan M. Boileau, Kayla Lenshoek, Tony T. Huang, Robert Blelloch

**Affiliations:** 1 The Eli and Edythe Broad Center of Regeneration Medicine and Stem Cell Research, Center for Reproductive Sciences, University of California, San Francisco, San Francisco, CA, USA; 2 Department of Urology, University of California, San Francisco, San Francisco, CA, USA; 3 Helen Diller Family Comprehensive Cancer Center, University of California, San Francisco, San Francisco, CA, USA; 4 Department of Biochemistry & Molecular Pharmacology, New York University School of Medicine, New York, NY, USA; 5 These authors contributed equally; 6 Present address: Department of Biomedical Engineering, Duke University, Durham, NC, USA; 7 Lead contact

## Abstract

Mammalian genomes replicate in a cell-type-specific order during the S phase, correlated to transcriptional activity, histone modifications, and chromatin structure. The causal relationships between these features and DNA replication timing (RT), especially during cell fate changes, are largely unknown. Using machine learning, we quantify 21 chromatin features predicting local RT and RT changes during differentiation in embryonic stem cells (ESCs). About one-third of the genome shows RT changes during differentiation. Chromatin features accurately predict both steady-state RT and RT changes. Histone H3 lysine 4 monomethylation (H3K4me1), catalyzed by KMT2C and KMT2D (KMT2C/D), emerges as a top predictor. Loss of KMT2C/D or their enzymatic activities impairs RT changes during differentiation. This correlates with local H3K4me1 loss and reduced replication origin firing, while transcription remains largely unaffected. Our findings reveal KMT2C/D-dependent H3K4me1 as a key regulator of RT and replication initiation, a role that likely impacts diseases associated with KMT2C/D mutations.

## In brief

Gökbuget et al. use machine learning to quantify chromatin state changes that predict DNA replication timing changes during cell differentiation. By following up on top predictors through functional validation, they uncover a new function of KMT2C/KMT2D-dependent H3K4me1 in regulating DNA replication timing changes and origin activity during ESC differentiation.

## Graphical abstract

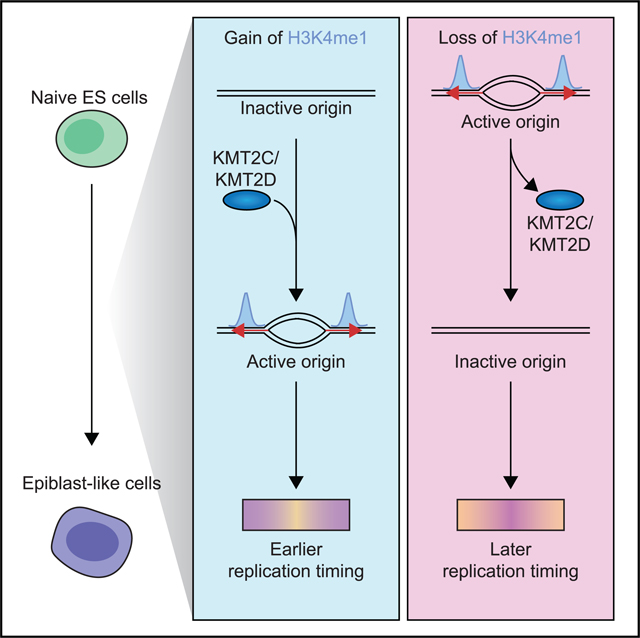

## INTRODUCTION

Chromatin exists at multiple structural levels, which integrate to modulate gene activity. Recent evidence suggests that the temporal order by which DNA is replicated during the S phase—known as DNA replication timing (RT)—is correlated with various chromatin features predictive of gene activity.^1–13^ Measurements of RT across the genome reveal megabase-scale segments with either early or late S-phase DNA replication (herein referred to as RT domains). On the level of chromatin structure, boundaries of frequently self-interacting domains defined by chromatin conformation capture assays—known as topologically associating domains (TADs)—align well with boundaries of RT domains,^14,15^ and specific TAD subtypes were recently implicated in replication initiation.^13^ Furthermore, early and late replicated RT domains correlate with higher-order chromatin compartmentalization into A and B compartments, respectively.^14,15^ Euchromatin-associated histone modifications are enriched in early-replicating RT domains,^10,16,17^ whereas heterochromatin-associated histone H3K9 methylation is enriched in late-replicating RT domains.^2^ Similarly, RT domains harboring actively transcribed genes are generally replicated earlier than ones with inactive genes.^3,5–9^ At the level of the whole nucleus, early-replicating or active chromatin localizes to the center of the nucleus, and late-replicating or repressed chromatin localizes to the periphery of the nucleus.^18–21^ While this evidence correlates RT with markers of gene activity, it remains unclear how these chromatin features causally and hierarchically relate to RT and how these relationships may change during cell state maintenance versus transition. Much of the evidence that correlates RT with selected chromatin features has been produced under steady-state conditions in unrelated cellular contexts, with functional evidence limited to selected genomic loci.^22,23^ These limitations complicate the comparability and consolidation of the data into a general model.

Here, we address these gaps by measuring genome-wide RT alongside genomic profiling of the chromatin state followed by machine learning to unbiasedly evaluate 21 chromatin features for their ability to locally predict RT within 50 kb genomic bins during the steady state and the transition of naive embryonic stem cells (ESCs) to epiblast-like cells (EpiLCs). We find that the local chromatin state accurately predicts local RT across the genome during the transition. Functional validation of the most predictive mark, H3K4me1, through genetic deletion of the responsible histone monomethyltransferases KMT2C/D (previously known as MLL3/4) resulted in genome-wide impairment of RT changes within smaller subdomains that normally occur during the transition (henceforth referred to as RT changes). This impairment was largely uncoupled from local changes in transcriptional activity. Furthermore, profiling of replication origin activity during the transition revealed a local requirement of KMT2C/D activity in promoting origin activity, which was most evident at sites of KMT2C/D-dependent H3K4me1 and RT changes. Overall, these data present a new role for KMT2C/D enzymatic activity in the regulation of DNA replication beyond its role in transcriptional regulation.

## RESULTS

### Genome-wide changes in RT during early ESC differentiation

**Figure 1.**
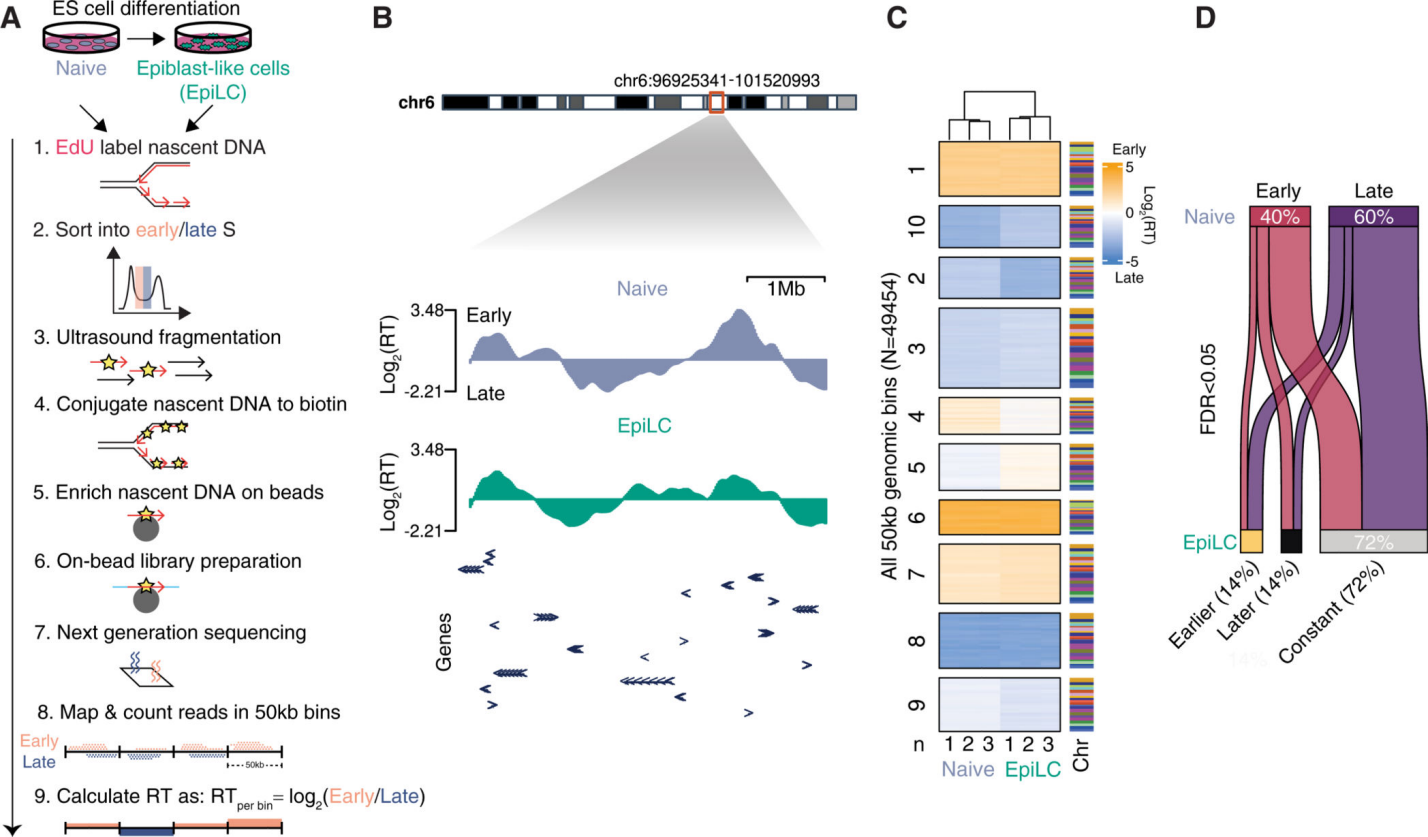
Genome-wide changes in replication timing during pluripotency transition (A) Scheme of BioRepli-seq method and analysis. (B) Genome track of log_2_ RT displaying early/late RT domains in naive (N) and EpiLC (Epi) states. Gene exons within the genome track are depicted with arrowheads. (C) Heatmap showing genome-wide RT for all 50 kb genomic bins per replicate of naive-to-EpiLC states. Associated chromosomes (chr) are shown. Clustering was performed by K-means. Individual replicates derived from three independent cultures for each cell state are shown. (D) Sankey plot summarizing all significant RT changes (FDR < 0.05) for all 50 kb genomic bins during naive-to-EpiLC transition.

To understand how RT is regulated during cellular differentiation, we used a previously established ESC model following the transition from the naive to the EpiLC state reflecting the physiologically relevant cell fate transition of the embryonic epiblast during the peri-implantation period of mammalian development (mouse embryonic day [E]4.5–E5.5).^24–27^ This EpiLC state is consistent with the recently hypothesized formative pluripotency state, which immediately precedes commitment to germ layer specifications.^28^ To measure genome-wide RT, we developed a fast and scalable EdU/biotin-labeling based approach (BioRepli-seq) based on the relative enrichment of sequenced nascent DNA within 50 kb genomic bins in early versus late S phase (Figure 1A). The strong biotin-streptavidin interaction allowed us to implement stringent washing steps under denaturing conditions, reduce cellular input, and perform on-bead library preparation, all of which constitute advantages over previous methods. Our results in naive ESCs correlated well with those using the two-fraction BrdU-based technique previously performed under the same culture conditions^29^ (Figure S1A). However, the BioRepli-seq method demonstrated an enhanced dynamic range (Figures S1A and S1B). Both naive and EpiLC states showed clearly delineated early- and late-replicating domains that change in size and RT across megabases during differentiation (Figure 1B). Principal-component analysis attributes ~80% of the variance in RT to the pluripotency state transition and confirms the reproducibility of the data within cellular states (Figure S1C). Heatmaps and statistical analysis of genome-wide RT changes during the pluripotency state transition reveal that ~30% of the genome changed RT (false discovery rate [FDR] < 0.05), with equal changes toward earlier and later RT (Figures 1C and 1D). Together, these results reveal extensive changes in RT genome-wide during the relatively short developmental time window reflected by the naive to EpiLC transition.

### Chromatin state accurately predicts RT under steady-state and differentiation conditions

**Figure 2.**
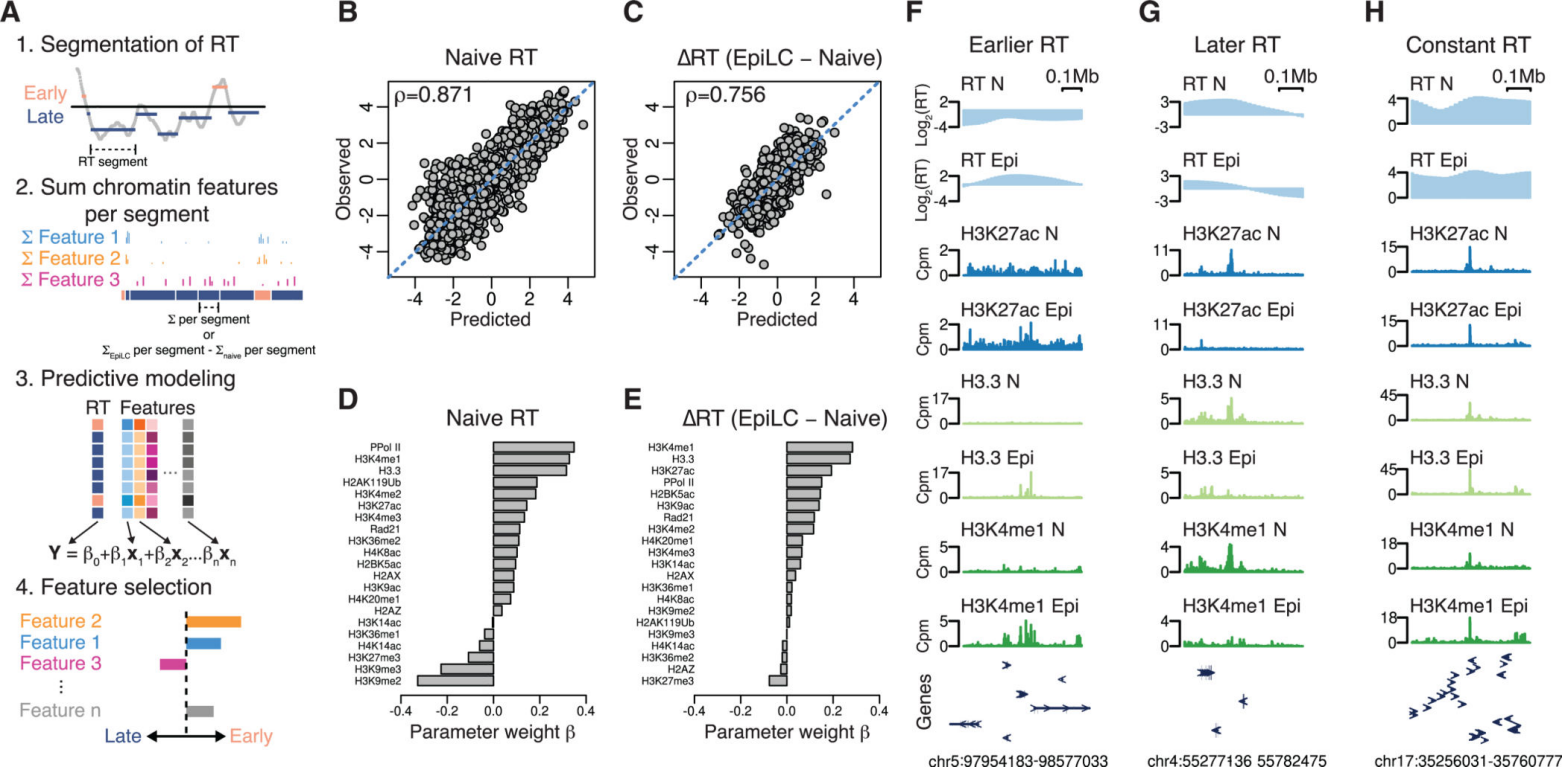
Chromatin state accurately predicts global RT, with H3K4me1 emerging as a top predictor (A) Workflow for RT segmentation, machine learning, and feature selection. (B and C) Scatterplots showing predicted versus observed naive RT (B) and ΔRT (C) during transition (difference of EpiLC and naive RT) based on fitted elastic net regression machine learning model. Spearman’s correlation coefficient (rho) is shown. (D and E) Parameter weight for each chromatin feature for naive RT (D) and ΔRT (E) during transition based on fitted elastic net regression machine learning model. A larger magnitude of parameter weight implies more predictive strength of feature. See STAR Methods for details. (F–H) Example genome tracks of regions showing earlier (F), later (G), and constant (H) RT and the associated chromatin feature signals of the top 3 predicting features (see D and E) in naive (N) and EpiLC (Epi) states. Exons are depicted with arrowheads.

Next, we investigated the association between the chromatin state and RT in steady-state naive pluripotency, as well as how alterations in the chromatin state are linked to changes in RT during the naive-to-EpiLC transition. To do so, we measured the genome-wide signal of 21 chromatin features including histone modifications (H3K4me1–3, H3K27ac, H3K27me3, H3K36me1–2, H3K9ac, H3K14ac, H3K9me2–3, H4K8ac, H4K16ac, H4K20me1, H2BK5ac, and H2AK119ub), histone variants (H3.3, H2A.Z, and γH2AX), chromatin architectural factors (cohesin complex), and actively transcribing RNA polymerase II (RNA Pol II; phosphorylated at S2/5) using cleavage under targets and tagmentation (CUT&Tag) under naive steady-state conditions and following the naive-to-EpiLC transition (Figures S1D and S1E). These data were processed and then used in elastic net regression models to determine which chromatin features were most predictive of naive RT and changes thereof during differentiation (Figure 2A; also see STAR Methods). Adjacent 50 kb genomic bins were grouped into segments of similar naive RT or delta (difference of EpiLC and naive state) RT (ΔRT) using circular binary segmentation^30^ (Figures S2A and S2B; also see STAR Methods). The resulting segments for naive RT and ΔRT had comparable median sizes of ~1 Mb, with 1,894 naive RT segments and 1,077 ΔRT segments in total (Figures S2C and S2D). For each chromatin feature, we identified peaks using sparse enrichment analysis for CUT&RUN (SEACR) and summed the background-adjusted peak signals within each RT segment (see STAR Methods). For ΔRT segments, we computed the difference in summed signals between the EpiLC and naive steady states for each segment. Consequently, we derived a matrix comprising either the combined chromatin feature peak signals in naive steady states or their signal differences during the transition for each RT segment across all 21 chromatin features. This dataset was subsequently assessed for its predictive capacity of steady-state naive RT and ΔRT during differentiation using elastic net regression. Elastic net regression, a regularized machine learning model, shrinks the weights of parameters that offer redundant or no information to the model fit, facilitating the selection of uniquely predictive chromatin features. The resulting models showed that chromatin features were collectively highly effective at predicting both steady-state naive RT and ΔRT during differentiation, as evidenced by strong correlations of predicted versus observed RT (Figures 2B and 2C).

To gain deeper insights into which specific chromatin features might be most influential on steady-state RT and RT changes, we examined the parameter weights of the individual features in the constructed regression models (Figures 2D and 2E). In the case of naive RT, our analysis revealed strong positive parameter weights for phosphorylated RNA Pol II, H3K4me1, and H3.3, along with strong negative weights for H3K9me2/3 and H3K27me3. For ΔRT during differentiation, changes in H3K4me1, H3.3, and H3K27ac showed strong positive parameter weights, with only H3K27me3 showing a moderate negative parameter weight. The strong associations of H3K4me1, H3.3, H3K27ac, and RT were also readily apparent through visual examination of representative genome tracks (Figures 2F–2H).

To further validate the results of the elastic net regression model, we conducted linear regression for individual chromatin features and each pairwise combination. Correlation plots of genome-wide 50 kb bins showed the strong association of H3K4me1, H3.3, and phosphorylated RNA Pol II signal intensity with early or, in the case of differentiation, earlier RT (Figures S3A and S3B). Consistent with the elastic net regression models, linear regression analysis identified H3.3 and H3K4me1 as showing the strongest associations with both naive RT and changes in RT (see R^2^ values in Figures S3A and S3B). Specifically, H3.3 explained 56% and 32% of the variance in naive RT and RT changes, respectively, while H3K4me1 explained 55% and 27%, respectively (Figures S3A and S3B). Furthermore, pairwise linear regression analysis of all marks demonstrated that the combination of H3.3 and H3K4me1 outperforms their individual predictive strength, validating that each mark provides separate information (Figures S3C and S3D). Similarly, both marks provide independent information from phosphorylated RNA Pol II. In sum, these results provide quantitative evidence for H3K4me1 and H3.3 as the strongest predictors of early/earlier RT in the steady state and during the naive-to-EpiLC transition. In contrast, while the repressive marks H3K9me2/3 and H3K27me3 are predictive of late RT in naive ESCs, only H3K27me3 is moderately predictive of a shift to later RT during the transition.

### KMT2C/D activity is required for genome-wide RT changes during ESC differentiation

**Figure 3.**
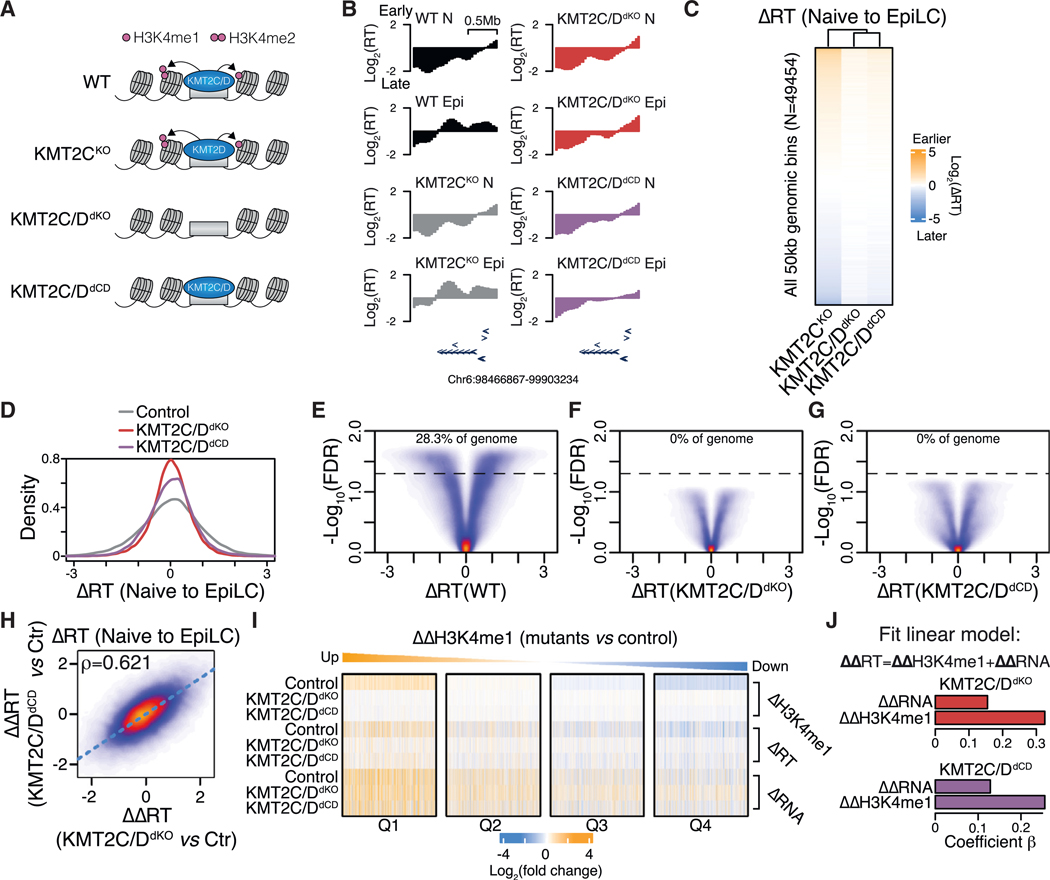
KMT2C/D activity shapes genome-wide RT dynamics during ESC differentiation (A) Scheme of loss-of-function models. (B) Example genome track of KMT2C/D activity-dependent RT domain showing average RT (derived from three independently grown cultures) of WT, KMT2C^KO^, KMT2C/D^dKO^, and KMT2C/D^dCD^ ESCs in naive (N) and EpiLC (Epi) states. (C and D) Heatmap (C) and density plot (D) showing genome-wide ΔRT (difference of EpiLC and naive RT) during cell state transition for control (KMT2C^KO^), KMT2C/D^dKO^, and KMT2C/D^dCD^ ESCs. Heatmap is sorted by control ΔRT. (E–G) Volcano plots showing genome-wide ΔRT versus negative log_10_(FDR) during naive-to-EpiLC transition for WT (E), KMT2C/D^dKO^ (F), and KMT2C/D^dCD^ (G) ESCs. The percentage of genome changing is shown for FDR < 0.05 (dashed line). (H) Correlation plot of changes in ΔRT in KMT2C/D^dKO^ relative to controls compared to KMT2C/D^dCD^ relative to controls. Spearman’s correlation coefficient (rho) is shown. (I) Heatmap showing differential (EpiLC relative to naive state) H3K4me1, RT, and transcription during transition for control (KMT2C^KO^), KMT2C/D^dKO^, and KMT2C/D^dCD^ ESCs for all 50 kb bins stratified into quartiles by changes in peak signal of H3K4me1 in controls during transition. (J) Coefficients of linear regression explaining difference in ΔRT between KMT2C/D^dKO^ (top) or KMT2C/D^dCD^ (bottom) relative control (KMT2C^KO^) using respective differences in H3K4me1 and transcription.

H3K4me1 was among the best overall predictors of early steady-state naive RT and earlier RT changes during differentiation, yet a causal role for this histone modification or the responsible enzymes in the regulation of RT remains unexplored. H3K4me1 is commonly thought of as a marker of either primed or active enhancers that control the expression of their cognate genes.^31^ The KMT2C/D enzymes are responsible for the H3K4 mono- and dimethylation at many of these enhancers,^32,33^ with KMT2D being the predominantly active isoform in ESCs.^34,35^ Surprisingly, the loss of KMT2C/D has little impact on the establishment of the EpiLC transcriptional program and protein marker expression during ESC differentiation.^35^ Furthermore, the point mutation of the catalytic site of these enzymes resulting in the loss of their H3K4 methyltransferase activity has little to no impact on the transcriptional state in the naive steady state^36^ or on transcriptomic changes during ESC differentiation.^35^ Hence, it remains unclear how these enzymes and their activity causally impact chromatin. We hypothesized that the regulation of RT may be one such role. To address this hypothesis, we performed BioRepli-seq in ESCs lacking KMT2C (KMT2C knockout [KMT2C^KO^]), both KMT2C and KMT2D (KMT2C/D double KO [KMT2C/D^dKO^]), or KMT2C and KMT2D catalytic activities (KMT2C/D^dCD^) under both steady-state naive conditions and following the naive-to-EpiLC transition (Figures 3A and S4A–S4C). Principal-component analysis confirmed the reproducibility of these data across three replicates, with a clear separation between cell state and genotype. KMT2C^KO^ overlapped with the wild type (WT), consistent with a primary role for KMT2D in these cells (Figures S4D and S4E).

Representative genome tracks showed that sites where RT normally shifts earlier during the naive-to-EpiLC transition in WT and KMT2C^KO^ cells failed to do so in KMT2C/D^dKO^ and KMT2C/D^dCD^ (Figure 3B). To expand on this analysis, we compared genome-wide changes in RT across all 50 kb bins between the naive and EpiLC states. Heatmap visualization of bins sorted by their RT change from earlier to later in KMT2C^KO^ showed strongly diminished RT changes in the two double-mutant ESC lines (Figure 3C). This diminishment was also evident in the reduced dynamic ranges of ΔRT in the double-mutant ESCs (Figure 3D). Indeed, while in controls, ~30% of the genomic bins showed significant changes in RT during the transition (FDR < 0.05), KMT2C/D^dKO^ and KMT2C/D^dCD^ were almost devoid of such changes (Figures 3E–3G). This dramatic loss of significant changes could not be explained by the high variability among the mutant versus control replicates, as the principal-component analysis showed strong alignment between the replicates in all states (Figures S4D and S4E). In addition, the reduction in the dynamic range of ΔRT in mutants during the naive-to-EpiLC transition (Figure 3D) was not evident in the naive and EpiLC steady states, arguing against a general reduction in the RT dynamic range upon KMT2C/D inactivation (Figures S4F and S4G). Furthermore, the deficiencies of KMT2C/D^dKO^ and KMT2C/D^dCD^ in ΔRT relative to controls during the naive-to-EpiLC transition were highly correlated (Figure 3H). These data uncover a causal role for the KMT2C/D proteins and their enzymatic activity in mediating dynamic changes in RT during early ESC differentiation.

### KMT2C/D activity is locally coupled to RT but largely uncoupled from transcription

The identification of KMT2C/D as a regulator of RT dynamics led us to investigate the extent to which these changes in RT may be linked to the loss of H3K4me1 and transcriptional dysregulation. To address this, we compared changes in H3K4me1, RT, and RNA levels during the naive-to-EpiLC transition in KMT2C/D^dKO^, KMT2C/D^dCD^, and control ESCs. We separated RT bins into quartiles based on the difference in H3K4me1 changes in controls compared to mutants (ΔΔH3K4me1) during the transition (Figure 3I). Quartile 1 and quartile 4 represented RT bins that show the greatest failure in gaining and losing H3K4me1; they similarly showed a clear impairment to change RT toward earlier and later timing, respectively. In contrast, changes in RNA levels showed little to no association with either H3K4me1 or RT at most RT bins. Consistent with these observations, linear regression analysis across all genomic bins showed that changes in H3K4me1 in KMT2C/D^dKO^ or KMT2C/D^dCD^ were much more predictive of changes in RT than changes in RNA levels (Figure 3J). To evaluate what other chromatin features are associated with KMT2C/D-dependent H3K4me1 peaks, we compared the CUT&Tag signals for all 21 evaluated features at dependent and independent H3K4me1 peaks (Figure S4H). This analysis revealed that dependent peaks were enriched for the highest levels of H3K4me1, H3K27ac, and H3.3 but reduced the levels of the promoter feature H3K4me3 and the repressive modification H3K27me3, consistent with KMT2C/D-dependent H3K4me1 being found primarily at enhancers.

We extended our transcriptional analysis to naive steady-state conditions. Out of 3,701 genomic bins that lose H3K4me1 upon loss of KMT2C/D proteins or their catalytic activity, 6%–7% showed a significant change (FDR < 0.05) in RT in KMT2C/D^dKO^ and KMT2C/D^dCD^ versus control KMT2C^KO^ naive ESCs (Figures S4I–S4O). We then again compared and clustered changes in H3K4me1, RT, and transcription at KMT2C/D-dependent H3K4me1 genomic bins between the mutants and the control, resulting in five distinct clusters. Two clusters showed an association between the loss of H3K4me1 and a shift to more consistently later RT (Figure S4P, see clusters 2 and 5). Of these, only the smaller one (cluster 2)—representing ~2.5-fold fewer genomic bins (309 versus 782 bins)—showed an association with changes in RNA levels. Linear regression across all genomic bins showed associations with both but to a larger degree between RT and H3K4me1 than between RT and transcription (Figure S4Q). Collectively, these data reveal that KMT2C/D-dependent H3K4me1 locally coordinates RT dynamics during the naive-to-EpiLC transition, which largely happens independent of transcriptional regulation.

### KMT2C/D-dependent H3K4me1 locally associates with DNA replication origin activity

**Figure 4.**
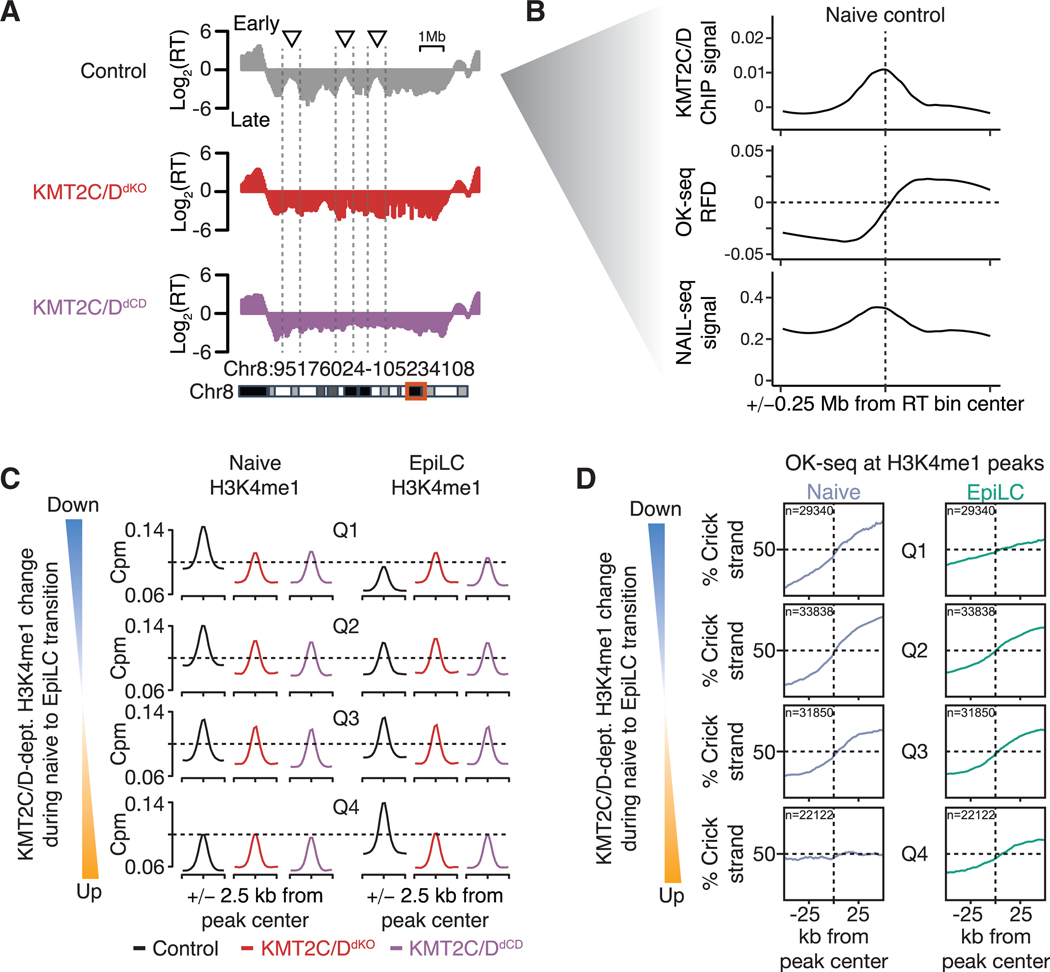
KMT2C/D activity locally associates with replication origin firing (A) Example genome track of KMT2C/D-dependent naive RT domain for KMT2C^KO^ (Control), KMT2C/D^dKO^, and KMT2C/D^dCD^ highlighting the loss of early peaks within larger later domains. (B) Metagene analysis of naive WT KMT2C/D ChIP-seq (counts per million [cpm] of WT subtracted by KMT2C/D^dKO^ negative control), naive WT OK-seq replication fork direction (RFD), and naive WT NAIL-seq (RPKM) centered at all KMT2C/D-dependent naive RT domains (changing more than 2-fold on the log_2_ in mutants versus control) that also show loss of H3K4me1 (lost in mutants by more than 1-fold on log_2_ scale). (C and D) Metagene analysis of H3K4me1 CUT& RUN (C) and OK-seq (D) data at H3K4me1 peaks stratified into quartiles (Q1–Q4) based on their KMT2C/D-dependent H3K4me1 read countchange in control versus mutants (average of changes in KMT2C/D^dKO^ and KMT2C/D^dCD^) during naive-to-EpiLC differentiation. Q1 and Q4 represent sites with the greatest gain and loss of KMT2C/D-dependent H3K4me1, respectively.

To gain further mechanistic insights into how KMT2C/D locally regulate DNA replication, we analyzed genomic regions affected by KMT2C/D loss at the RT and H3K4me1 levels. Genome tracks revealed that these regions appeared as smaller, relatively earlier-replicated peaks with mid-S-phase RT within mega-base-scale, late-S-phase-replicating RT domains (Figure 4A). Loss of KMT2C/D or their enzymatic activities resulted in the disappearance of these peaks, transforming them into large, consistently late-replicating domains. Based on this pattern, we hypothesized that KMT2C/D activity might promote the relatively earlier RT associated with these peaks by regulating local replication origin activity.

To test our hypothesis, we first evaluated existing KMT2C/D chromatin immunoprecipitation sequencing (ChIP-seq)^36^ and Okazaki fragment (OK)-seq^37^ datasets derived from naive ESCs. OK-seq maps OKs in a strand-specific manner to identify replication origins based on Watson-Crick strand bias asymmetry. Metagene analysis showed that KMT2C/D binding and Okazaki strand bias asymmetry were colocalized within KMT2C/D-dependent RT peaks. These data show the presence of replication origins at or near KMT2C/D binding sites (Figure 4B). Consistent with the OK-seq data, another method of identifying replication origins, nucleoside analog incorporation locus (NAIL)-seq,^17^ also showed colocalization with KMTC/D signals (Figure 4B). Extension to other marks using elastic regression showed that of the 21 chromatin features evaluated, H3K4me1 was the most predictive of replication origin sites (Figure S5A). These analyses show that KMT2C/D and its enzymatic product H3K4me1 locally associate with and are predictive of replication origins within regions of KMT2C/D-dependent RT in steady-state naive ESCs.

Next, we performed our own OK-seq to evaluate the genome-wide association between changes in H3K4me1 and changes in origin firing at the H3K4me1 sites during the naive-to-EpiLC transition. Prior work in immortalized RPE-1 cells has shown an enrichment for origin firing at transcriptional start sites (TSSs), which correlates with gene activity.^38^ Therefore, we performed metagene analysis on two biological OK-seq replicates at TSSs separated into quartiles based upon the expression levels of the cognate genes in both the naive cells and EpiLCs (Figure S5B). The resulting plots showed a strong correlation between origin firing efficiency (as defined by strand bias asymmetry) and transcriptional activity, with most efficiently firing origins at highly transcribed TSSs. These data support prior work uncovering origins at TSSs, which correlate with gene expression^38–40^ but now in a developmental context. They also validate the quality of our OK-seq libraries.

To next evaluate a potential association of origin activity and KMT2C/D activity, we first stratified all H3K4me1 peaks into quartiles based on the change in H3K4me1 read counts during differentiation between mutants and controls (ΔH3K4me1 control — ΔH3K4me1 mutants). Quartile 1 represents peaks that exhibited the greatest loss in KMT2C/D-dependent H3K4me1 during differentiation, whereas quartile 4 represents peaks that showed the greatest gain in signal (Figure 4C). Metagene analysis of the OK-seq centered on these peaks showed a striking association between the H3K4me1 signal and OK-seq strand bias asymmetry (Figure 4D). That is, sites that lose H3K4me1 during differentiation also show a reduction in OK strand bias (quartile 1), whereas sites that gain H3K4me1 during differentiation associated with increased strand bias (quartile 4). This association was confirmed through analysis of published OK-seq, NAIL-seq, and small nascent strand (SNS)-seq^41^ data (Figures S5C–S5E). Together, these data reveal a genome-wide association between KMT2C/D-dependent H3K4me1 and local origin firing efficiency.

### KMT2C/D-dependent H3K4me1 promotes DNA replication origin firing

Given the striking association between KMT2C/D-dependent H3K4me1 and replication origin efficiency, we next asked how the loss of KMT2C/D activity impacts origin firing. To do so, we performed OK-seq in KMT2C/D^dKO^ and KMT2C/D^dCD^ cells in both the naive ESCs and EpiLCs. Again, the quality of the libraries was confirmed by evaluating TSSs stratified into quartiles by gene expression (Figure S5B). As expected TSSs of the highest transcribed genes showed the greatest strand bias asymmetry, as seen in controls. Importantly, these asymmetries seen in the KMT2C/D^dKO^ and KMT2C/D^dCD^ mutants were indistinguishable from controls, showing that KMT2C/D activity does not regulate origin firing at TSSs (data not shown).

**Figure 5.**
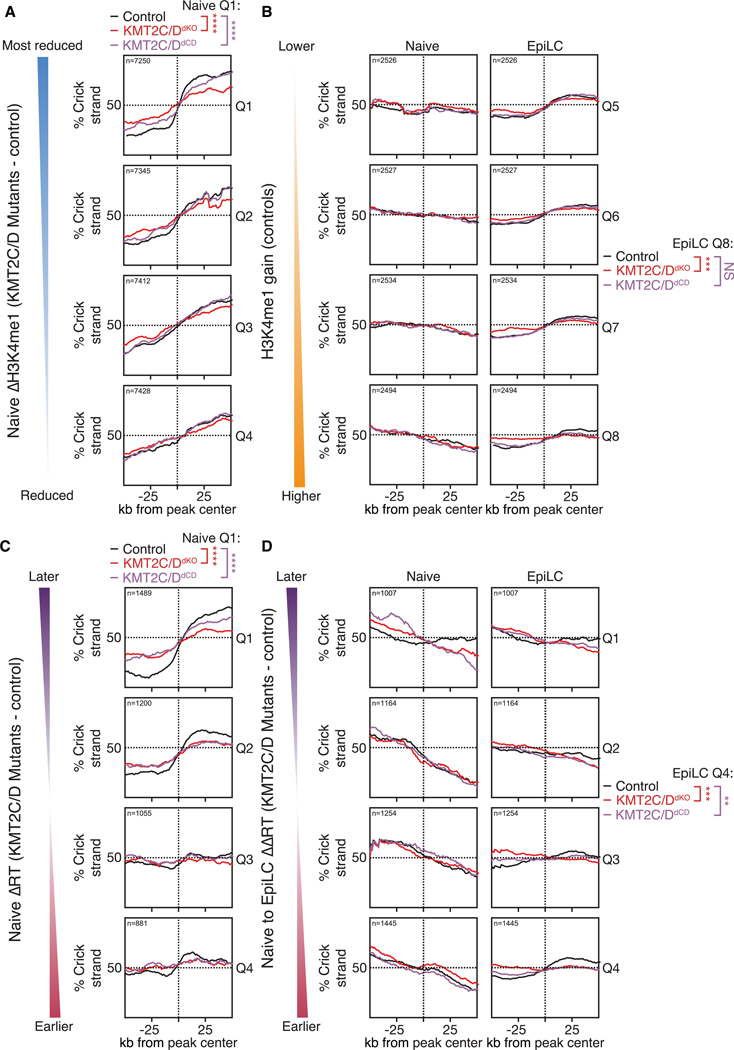
KMT2C/D activity locally promotes replication origin firing (A) Metagene analysis of OK-seq data from control (KMT2C^KO^), KMT2C/D^dKO^, and KMT2C/D^dCD^ cells at KMT2C/D-dependent H3K4me1 peaks in naive state. Peaks were stratified into quartiles (Qs) based on their H3K4me1 read count change in mutants (average of KMT2C/D^dKO^ and KMT2C/D^dCD^) versus control. KMT2C/D-dependent peaks showing more than 2-fold reduction in H3K4me1 in mutants versus control were used as input. (B) Metagene analysis of OK-seq data from control (KMT2C^KO^), KMT2C/D^dKO^, and KMT2C/D^dCD^ cells at gained H3K4me1 peaks during differentiation. Peaks were stratified into 8 Qs based on their H3K4me1 read count change in controls during the naive-to-EpiLC differentiation (see Figure S5F for Q1–Q4). Only gained peaks showing more than a 2-fold gain in H3K4me1 in controls were used as input. (C) Metagene analysis of OK-seq data from control (KMT2C^KO^), KMT2C/D^dKO^, and KMT2C/D^dCD^ cells at KMT2C/D-dependent H3K4me1 peaks in naive state. Peaks were stratified into Qs based on their RT change in mutants (average of KMT2C/D^dKO^ and KMT2C/D^dCD^) versus control. KMT2C/D-dependent peaks showing more than a 2-fold reduction in H3K4me1 in mutants versus control were used as input. (D) Metagene analysis of OK-seq data from control (KMT2C^KO^), KMT2C/D^dKO^, and KMT2C/D^dCD^ cells at gained H3K4me1 peaks during differentiation. Peaks were stratified into Qs based on their difference in RT changes in controls versus mutants (average of KMT2C/D^dKO^ and KMT2C/D^dCD^) during the naive-to-EpiLC differentiation. Only gained peaks showing more than a 2-fold gain in H3K4me1 in controls were used as input.

Next, to investigate whether origins at KMT2C/D-dependent H3K4me1 peaks are affected, we first stratified these peaks into quartiles based on the degree of H3K4me1 loss in mutants compared to controls for the naive state. Analysis of OK-seq data at these KMT2C/D-dependent peaks in naive cells showed that peaks with the greatest loss in H3K4me1 (quartile 1) exhibited the largest strand bias in control cells (Figure 5A, black lines). Both the loss of KMT2C/D and their enzymatic activities significantly reduced the strand bias, with the greatest impact in quartile 1, uncovering a causal relationship between KMTC/D methyltransferase activity and origin firing efficiency at the KMT2C/D-dependent H3K4me1 sites in naive ESCs.

Next, we evaluated peaks that typically gain H3K4me1 in control samples during differentiation. Peaks exhibiting at least a 2-fold increase in H3K4me1 were stratified into octiles, ranging from the lowest to the highest H3K4me1 increase. Analysis of OK-seq data centered on these peaks in each octile revealed a clear gain in strand bias between the naive and EpiLC states (Figures 5B and S5F). However, only the top octile (octile 8), which represents the greatest increase in H3K4me1 from naive to EpiLC states, showed a significant reduction in strand bias in the OK-seq data in KMT2C/D^dKO^ cells versus controls. KMT2C/D^dCD^ exhibited a trend toward reduction, but it was not statistically significant. The smaller effect observed in the transition data is likely due to unavoidable heterogeneity in differentiation rates among cells in the culture dish.

To address the relationships between H3K4me1, RT, and origin firing, we stratified KMT2C/D-dependent H3K4me1 peaks into quartiles based on the change in RT, considering only bins containing those peaks, in KMT2C/D mutant compared to control cells. First, we focused on naive cells (Figure 5C). Quartile 1 represents the greatest delay, while quartile 4 represents the least delay in RT in mutants compared to controls. Origin firing at the KMT2C/D sites was strongly associated with the impact of the mutants on RT (Figure 5C, black lines). That is, sites in the most-impacted RT bins (quartile 1) showed the greatest strand bias asymmetry, while those in the less-impacted bins (quartiles 3 and 4) showed little asymmetry. Furthermore, the KMT2C/D^dKO^ and KMT2C/D^dCD^ cells showed a highly significant reduction in origin firing, as seen by reduced asymmetry, in the most highly impacted RT bins (quartiles 1 and 2, red and purple lines). Thus, a loss in KMT2C/D activity in naive ESCs leads to a simultaneous reduction in origin firing and a delay in RT, specifically at regions with KMT2C/D-dependent H3K4me1.

Next, we assessed how the disruption of RT in bins harboring KMT2C/D-dependently gained H3K4me1 peaks during the naive-to-EpiLC transition impacted origin firing. Peaks were stratified into quartiles based on the directionality and degree of their RT change during the transition. Quartile 1 represents sites that show the greatest delay in RT, while quartile 4 represents the greatest advancement in RT during the naive-to-EpiLC transition. Notably, bins with most advanced RT (quartile 4) during the transition showed a gain in strand bias asymmetry (Figure 5D, black lines), indicating an association between earlier RT and increased origin firing. This increase in origin firing was significantly diminished in the KMT2C/D mutants (red and purple lines). Together, these data show that KMT2C/D activity is simultaneously promoting origin firing and earlier RT, which is most evident at sites that display the greatest defects in H3K4me1 and RT in mutant cells. These results uncover a functional role of H3K4 monomethylation, catalyzed by KMT2C/D, in promoting origin firing, which in turn is likely responsible for the advancement of RT during the S phase at the same sites, both in the steady state and during differentiation.

## DISCUSSION

Our results present a previously unknown role for KMT2C/D histone monomethyltransferases in locally coordinating H3K4me1 deposition with replication origin firing and RT. In the absence of KMT2C/D proteins or their enzymatic activities, genomic regions that normally exhibit dynamic H3K4me1 during the pluripotency cell state transition show a coordinated loss of H3K4me1 and a corresponding local impairment in RT dynamics. These regions of dynamic H3K4me1 positively correlated with local origin firing, which is impaired upon the loss of KMT2C/D or their enzymatic activities. Therefore, these findings uncover functions for KMT2C/D and H3K4me1 that are beyond their canonical roles in transcriptional regulation and almost certainly underlie, in part, developmental defects and disease phenotypes associated with loss-of-function mutations in these genes.

Our machine learning approach builds on previous correlative analyses that have associated active chromatin features with early RT and inactive chromatin features with late RT.^2,3,14,17,42,43^ It does so by unbiasedly addressing how much independent information each chromatin feature, under identical cellular conditions, provides toward the prediction of RT. Additionally, we apply this approach to a developmentally relevant cell state transition to determine the extent to which changes in chromatin features can predict changes in RT. Previously, predictive modeling across three fly cell lines identified H3K4me2, among other chromatin features, as a top predictor of replication origin locations, suggesting potential broad conservation of the mechanism.^44^ Another one of our hits, H3.3, was recently demonstrated to play a crucial role in the timing of early replication domains under the steady state in the cell line HEK293.^45^

KMT2C/D are part of a larger family of KMT2/MLL proteins that are canonically thought to be directly involved in transcriptional regulation through deposition of active chromatin marks H3K4me3 at promoters by KMT2A/B and H3K4me1 at enhancers by KMT2C/D.^31,46^ While the proteins and their homologs are essential for embryogenesis and cell growth across multiple species,^47–52^ the developmental requirement of their enzymatic activity seems to be more species specific. In flies, the loss of H3K4 monomethyltransferase activity by mutation of the enzymatic domain of Trr is compatible with development and results in minimal gene expression changes.^53^ However, mutant mice expressing enzymatically inactive KMT2C/D die during early development at embryonic day 6.5.^50^ While these lethal phenotypes were linked to changes in transcription, our data indicate that these changes might be a result of defects in replication initiation and RT. In particular, defects in RT are associated with DNA damage,^54^ which could be a key driver of early embryonic lethality^50,55^ and preceding cellular defects^56^ upon *Kmt2c/d* disruption.

We see little evidence for a correlation between KMT2C/D-driven changes in RT and gene expression changes. Similar to prior findings,^35^ we do not see a correlation between H3K4me1 loss and local transcriptional changes upon inactivation of KMT2C/D. Enhancers can act at a distance, and therefore, we may be occasionally correlating them with the wrong target gene. However, Perturb-seq studies show that most enhancers act locally, targeting the nearest TSS,^57,58^ and thus should be reflected in our genome-wide analyses. In either case, we find the effect of KMT2C/D-dependent H3K4 monomethylation on origin firing and RT to be local. Furthermore, while we confirm the presence of origins at TSSs whose efficiency correlates with transcription,^38^ these origins are not impacted by the loss of KMT2C/D or their methyltransferase activity. This suggests that there are different types of origins with distinct mechanisms of control and likely varying impacts on RT. For instance, RIF1, one of the few known regulators of RT, affects RT domains globally.^59–62^ In contrast, KMT2C/D-dependent activation of local replication origins results in earlier RT within specific subdomains but not across entire domains. This localized effect is likely due to compensation by other KMT2C/D-independent origins. Additionally, we cannot exclude the possibility that the relatively earlier RT associated with KMT2C/D-dependent origins represents stochastic local RT rather than consistently earlier regional RT. Single-cell origin firing and RT approaches are needed to delineate the relationship between local origin firing and RT genome wide. Moreover, although RIF1 impacts RT globally, it preferentially binds within late-replicating chromatin. Its global effect on RT is proposed to result from the derepression of late-replicating origins, leading to competition for replication initiation factors with early-replicating origins, thereby delaying their replication.^62^ Overall, these results highlight a complex interplay between regulators of origin activation and the availability of initiation factors in establishing cell-type-specific RT, which needs to be further investigated. Additionally, it will be important to explore the developmental progression of subdomains that display RT changes beyond ESC differentiation and whether they may act as a seed for domain-wide RT changes in downstream lineages.

Similar to our study, others have shown a genome-wide association between H3K4me1 and origin firing in steady-state immortalized mouse and human cell lines.^17,40,63^ However, in our study, we expand on this knowledge by evaluating how this association correlates with changes in origin firing during cellular differentiation and, importantly, by using KMT2C/D mutants, showing that the two can be causally linked. Whether the impact of KMT2C/D-dependent H3K4me1 on replication origin firing is a result of the direct recruitment of replication machinery or an indirect result of rendering the local chromatin environment more permissive to recruitment of such machinery remains to be determined. H3K4me1-associated proteomes^64^ and fork proteomes^65^ derived from different mammalian cell types show significant overlaps, suggestive of a potential role in recruitment. Further evidence is needed to distinguish between a direct and indirect permissive role for KMT2C/D in promoting origin firing.

Spatial genome organization is known to closely relate to genome-wide RT at various levels of resolution.^1^ Microscopy data indicate that simultaneously actively replicating sections of DNA structurally coalesce microscopically as replication foci,^66,67^ whereas chromatin conformation capture genomics techniques identified RT domains to show frequent intramolecular three-dimensional (3D) interactions.^2,11,14,15^ Recent studies have provided functional evidence for a subset of 3D chromatin loops in locally promoting early RT^16^ and defining local initiation zones.^13^ Intriguingly, KMT2C/D-dependent H3K4me1 can facilitate cohesin recruitment.^68^ Therefore, it is possible that KMT2C/D activity promotes replication origin firing by enhancing 3D chromatin interactions (Figure S5G). Temporally, this mechanism is feasible because H3K4me1 is largely retained during mitosis.^69,70^ Consequently, H3K4me1 could act upstream or synergistically with 3D chromatin interactions and RT, which are established after mitosis in early G1. Additionally, structurally related H3K4 methyltransferases have been shown to possess mitotic bookmarking functions,^71^ which could be linked to the role of KMT2C/D in regulating replication origins that we have discovered. A recent study in active B cells under steady-state conditions showed that altering RT by MCM6 knockdown does not lead to major changes to higher-order 3D chromatin interactions.^72^ Additionally, during mouse fertilization and development, defined temporal and spatial patterns of DNA replication can be detected in pronuclei and zygotes^67,73^ preceding 3D chromatin organization into TADs, which are gradually manifested following the first cell division.^74–76^ Furthermore, 3D chromatin organization changes are only weakly correlated with RT changes during the first cell cycle of differentiating human ESCs.^43^ Thus, RT and 3D chromatin interactions can operate independently. More studies are required to understand how the various levels of spatial genome organization causally influence RT and origin firing and how KMT2C/D may be involved.

*KMT2C* and *KMT2D* are both among the 10 most mutated genes in cancer (20% of all cancers).^77^ Our results present KMT2C/D as one of a few known regulators of RT, expanding its functional spectrum beyond transcriptional regulation. Whether changes in RT or origin firing could explain, in part, the close association of mutations of KMT2C/D with cellular transformation will be important to investigate. Together, our results elucidate the local functional relationship of the epigenome with RT that can, at least in the context of KMT2C/D regulation, be uncoupled from transcription and linked to the activation of replication origins. As such, our study offers an important contribution to understanding how chromatin state shapes essential cellular functions.

### Limitations of the study

In addition to the open questions discussed above, there are other limitations to our study. One such limitation is the interpretation of the absence of a signal in our machine learning approach. While predictive modeling is a powerful tool to identify candidate regulators of RT, the lack of predictive strength does not rule out a chromatin feature’s function in RT. For example, while H2A.Z has been implicated in promoting earlier RT in the cancer cell line HeLa, it did not emerge as a strong predictor in our models,^78^ indicating that its impact on RT may be cellular-context dependent or diluted by its association with genomic regions that do and do not change RT. Additionally, limitations in the CUT&Tag technology, including the quality of antibodies, can negatively impact signals for certain features. Furthermore, there are many other chromatin features, some of which almost certainly have important functional roles in RT and origin regulation, that were not evaluated here. Another limitation is the strength and resolution of the RT and OK-seq signal, which can be negatively impacted by several factors, including any heterogeneity in the cell population, especially during a cell fate transition; the resolution of S-phase fractions; the stochastic nature of origin firing; and the potential regional rather than discrete effects of H3K4me1 on origin firing. While we do not believe that these limitations impact our conclusions, their resolution through future studies and technological advancements is certain to provide further insight into the remarkable coordination of RT changes with cell fate transitions.

## STAR★METHODS

### RESOURCE AVAILABILITY

#### Lead contact

Further information and requests regarding resources and reagents should be directed to and will be fulfilled by the lead contact, Robert Blelloch (robert.blelloch@ucsf.edu).

#### Materials availability

All reagents generated in this study are available upon request.

#### Data and code availability

- Raw and processed BioRepli-seq, CUT&Tag, and OK-seq data have been deposited at NCBI GEO and are publicly available as of the date of publication. Accession numbers are listed in the key resources table.
- Original code used in this study is publicly available through GitHub as of the date of publication. The link is available in the key resources table.
- Any additional information required to reanalyze the data reported in this paper is available from the lead contact upon request.

### EXPERIMENTAL MODEL AND STUDY PARTICIPANT DETAILS

#### Cell culture

Mouse embryonic stem (ES) cells were grown at 37°C/5% CO_2_ in KO DMEM with 15% FBS, 1× NEAA, 1× L-Glutamine, 1× Penicillin/Streptomycin in the presence of LIF and 2i (1 μM PD0325901 and 3 μM CHIR99021) on gelatinized cell culture plates. Medium was exchanged daily. KMT2D depletion in KMT2C^KO^/KMT2D^cKO^ ES cells^56^ expressing Cre^ER^ was induced by the addition of 1 μM 4-Hydroxytamoxifen (4HT) as previously described.^35^ Male wildtype ES cells (v6.5) were derived from a C57BL/6 × 129/sv cross background. Male KMT2D^cCD^ ES cells^36^ (R1) were derived from a 129 strain background. KMT2C^KO^/KMT2D^cKO^ ES cells (sex undetermined^56^) were derived from a mixed background of C57BL/6 and 129 strains. Cells were routinely tested negatively for mycoplasm contamination.

#### Naive to epiblast-like cell differentiation

Differentiation from naive to epiblast-like cell (EpiLC) state was performed as previously described^24^ and validated for all KMT2C/D loss-of-function ES cell lines.^35^ Briefly, 5 × 10^5^ V6.5 (WT), KMT2C^KO^, KMT2C/D^dKO^ or KMT2C/D^dCD^ were seeded on gelatinized 6 well plates. The next day, cells were washed with 1× PBS and grown in medium without Lif+2i for approximately 63 h with daily media changes. For naive control timepoint, additional cells were grown in parallel in medium with Lif+2i. Differentiation was validated by coexpression of miR-290 promoter-driven RFP and miR-302 promoter-driven GFP using dual-reporter ES cells (v6.5)^26^ grown in parallel. The miR-290 promoter is known to be expressed in naive ES cells and from E3.5 to E6.5 during mouse development, whereas the miR-302 promoter is inactive in the naive state and becomes active upon ES cell differentiation and in E5.5 epiblasts during development. Cells with coincident expression of GFP and RFP are defined as EpiLCs and have almost identical transcriptomic signatures as post-implantation E5.5 epiblast cells.^25–27^ Furthermore, EpiLC derived from this differentiation follows a similar transcriptomic trajectory as alternative protocols.^27^

### METHOD DETAILS

#### Biotin Repli-seq (BioRepli-seq)

EdU was added to medium at a concentration of 100 μM and cells were grown for 2 h at 37°C/5% CO_2_. Next, cells were washed with cold PBS and trypsinized for 3 min at 37°C followed by quenching with cold medium. Cells were pipetted through 35 μM cell strainers and then spun at 300×g for 3 min. Next, cell pellet was resuspended in 290 μL PBS and 700 μL −20°C cold EtOH was added dropwise while vortexing. Cells were fixed overnight at −20°C. The next day, cells were spun at 300×g for 5min, washed once with 1% FBS in PBS and then resuspended in PI/RNase for DNA staining. After 20 min incubation at RT, cells were placed on ice and 6×10^5^ cells were sorted (BD FACSAria) into early S and late S fractions based on stained DNA content profiles. Next, genomic DNA was extracted using the NucleoSpin Tissue kit according to manufacturer’s instructions and eluted in 100 μL H_2_O. 50 μL of sample was then sonicated for 90 s (Covaris S220) with the following settings: PIP: 175, duty factor: 10, CPB: 200, Temperature: 4°C. Following, sonication, 50 μL of sheared DNA was mixed with 6.5 μL 10xTBS, 0.65 μL biotin azide (1mM stock), 1.3 μL Cu/THPTA (obtained by 1:1 mixing 10 mM CuSO_4_ and 50 mM THPTA) and 6.5 μL Na-Ascorbate (100 mM fresh stock), and then incubated for 1 h at RT. Next, size selection and cleanup were performed using AMPURE beads. First, 0.5 eq of beads were added and incubated for 5 min at RT. After 2 min incubation on magnet tube rack, supernatants were transferred to fresh tubes, mixed with 0.4 eq beads and incubated for another 5 min. Beads were then washed twice after incubation on magnet tube rack using 700 μL freshly prepared 80% EtOH and then transferred to new tubes for storage at −20°C. Approximately, 50 ng of DNA resuspended in 50 μL H_2_0 was then used for streptavidin pulldowns. Magnetic streptavidin beads were washed twice in B&W buffer (5 mM Tris pH 7.5, 0.5 mM EDTA, 1 M NaCl, 0.05% Tween 20) and 1 eq of washed streptavidin beads were added in 2×B&W to the DNA. After 15 min incubation at RT, beads were washed twice with 55°C warm 2×B&W buffer and once with 0.1x TE after incubation on magnet tube rack. Washed beads were resuspended in 25 μL 0.1× TE. On-bead library prep was performed using the NEBNext Ultra II (NEB) kit according to manufacturer’s instructions with the following changes. Half reaction volumes were used, and AMPURE beads purification steps were replaced by above-described washes with B&W buffer and 0.1× TE buffer. After adapter ligation step and washes, beads were resuspended in 7.5 μL 0.1× TE and boiled at 98°C for 10 min (with lid set to 105°C). Supernatant was used for PCR amplification and subsequent washes according to manufacturer’s instructions. Libraries were quantified and quality controlled using the Agilent 4200 Tapestation with D1000 reagents and sequenced.

#### BioRepli-seq data analysis

BioRepli-seq data was analyzed as previously described.^89^ Briefly, after mapping to mm10 using bowtie2,^79^ genomic reads were summed into non-overlapping 50 kb bins and log_2_ ratios of the early versus late S phase sorted fractions were computed per bin for each sample. Loess smoothing was performed for each chromosome as described^89^ using the ‘loess’ R function with the ‘span’ parameter set to 300 kb per length of chromosome and resulting data were registered into bedGraph files. Resulting files were converted into BigWig files for genome track and metagene plotting using ‘bedGraphToBigWig’ function from ucsc-tools.^90^ Statistically significantly changing 50 kb RT bins were determined using three replicates derived from independent cultures per condition in a Student’s t test followed by Benjamini & Hochberg (*FDR*) multiple comparison correction. To capture changes in RT during ES cell differentiation or between genotypes, we calculated the difference in RT compared states. Spearman correlations were computed using the ‘cor’ function with the setting “method = spearman”. For EdU-seq metagene analysis, mapped reads of the early S phase fraction were converted into BigWig files and Cpm-normalized read counts across replicates were averaged using the ‘bamCoverage’ function of the deepTools2 suite.^82^

#### CUT&Tag

As input into CUT&Tag profiling, naïve-to-EpiLC differentiation was conducted using dual-reporter ES cells^26^ and validated using flow cytometry (see ‘Naïve to epiblast-like cell differentiation section’). CUT&Tag was performed as previously described with the following adaptations.^35,91^ Briefly, freshly trypsinized cells were bound to activated Concanavalin A beads at a ratio of 10^5^ cells/7μL beads in CR Wash buffer (20mM HEPES, 150mM NaCl, 0.5mM Spermidine with protease inhibitors added) at room temp for 10 min. Volumes corresponding to 10^5^ cells were aliquoted into skirtless 96 well plates. After magnetization and withdrawal of supernatant, cells were resuspended via multichannel pipette in permeabilization buffer^91^ with added primary antibodies and incubated overnight at 4°C. After brief centrifugation, samples were resuspended in 1:100 secondary antibody (Guinea pig anti-rabbit) at 4°C for 1 h. Next, samples were washed with 200 μL of wash buffer three times for 5 min each. Samples were then incubated for 1 h at 4°C with 50 μL of CT Dig300 wash buffer (20mM HEPES, 300mM NaCl, 0.01% Digitonin, 0.5mM Spermidine with protease inhibitors added) with an added 25 nM pA-Tn5. Tn5 was loaded with adapters as previously described.^91^ After Tn5 incubation, samples were washed three times with CT Dig300 wash buffer for 5 min each. Plates were covered with foil and tagmentation initiated for 1 h at 37°C in a thermocycler with 10 mM MgCl2. Immediately afterward 1.6 μL of 0.5 M EDTA, 1 μL of 10 mg/mL Proteinase K, and 1 μL of 5% SDS was added to each sample via multichannel pipette. The plate was then covered with foil and incubated at 55°C for 2 h in a thermocycler to denature Tn5 and solubilize tagmented chromatin. After incubation, samples were magnetized, and the supernatant was transferred to new wells where SPRI bead purification was performed to select all DNA fragment lengths larger than 100 bp. Samples were eluted in 0.1× TE and approximately half of each sample was used for library preparation using NEBNext HIFI Polymerase with previously reported i7 and i5 indices.^92^ We ran enough cycles for each target to have enough product to pool and sequence (on average 12–18 cycles). After amplification, libraries were purified using SPRI size selection to enrich for fragments >250 bp and eluted in 0.1× TE. Quality and concentration of libraries were determined by an Agilent 4200 Tapestation with D1000 reagents before pooling for sequencing at BGI using DNBSEQ-G400 technology.

#### OK-seq

Naive to EpiLC differentiation was scaled up to 15 cm dishes. At the day of collection, 5 mL of conditioned media was collected for each plate, kept at 37°C, mixed with 2 mL fresh media, and EdU was added (60 μM final concentration). Next, EdU-containing media was added to cells (25 μM final concentration) and after gentle swirling cell were incubated for 2 min. Media was aspirated and 5 mL of ice-cold PBS was immediately added. Cells were washed once more with ice-cold PBS, and then scraped off and frozen in ice-cold DNA lysis buffer (10 mM Tris pH 8, 25 mM EDTA, 100 mM NaCl) using liquid nitrogen. Downstream OK-seq sample and library prep from cell lysates, were performed as previously described.^84^ Briefly, cell lysates were incubated with Proteinase K and SDS overnight at 50°C. DNA from cell lysates was purified by two sequential phenol-chloroform-isoamyl alcohol extractions, precipitated with ammonium acetate and ethanol, washed with 70% ethanol, and resuspended in TE overnight at 4°C. Okazaki fragments were isolated through size fractionation using ultracentrifugation (122,000 x g at 20°C) of a 5–30% linear sucrose gradient generated on a Beckman Coulter Optima XE-100. Size distribution was verified using a TapeStation (Agilent) assay. DNA <200 bp was concentrated by column centrifugation, and EdU-labeled Okazaki fragments were biotinylated by click chemistry reaction (10 mM Tris-HCl pH 8, 2 mM CuSO_4_, 10mM sodium ascorbate prepared fresh, 2mM Biotin TEG Azide. Any contaminating RNA was hydrolyzed (250mM NaOH at 37°C for 30 min) and DNA as phosphorylated by T4 polynucleotide kinase reaction EdU-click DNA was captured on MyOne T1 Streptavidin Dynabeads. OK-seq libraries were adaptor ligated, PCR amplified, and primer-dimers were removed with SPRI beads. Libraries were analyzed by TapeStation and sequenced (pair-end 50bp) using the NovaSeq 6000 Illumina sequencing platform and SP100 flow cells.

#### CUT&Tag data analysis

CUT&Tag data was mapped and analyzed using the automated Nextflow pipeline nfcore/cutandrun^80^ as previously described.^35^ Briefly, this pipeline performs trimming using Trim Galore and paired-end mapping of reads to mm10 using bowtie2 followed by filtering out low-quality reads and reads mapping to mm10 blacklist regions. Subsequently, the pipeline calls peaks using SEACR^81^ with a peak threshold of 0.05 and outputs their chromosomal coordinates and normalized signal contained within these coordinates. SEACR identifies peaks from sparse data (such as CUT&Tag) in which the background is largely consisting of “zeroes”. Next, consensus peak locations were generated based on peaks shared across experimental replicates. For genome track and metagene plots, mapped BAM files merged by replicates generated by the pipeline were converted to counts per million (Cpm)- normalized BigWig files using the ‘bamCoverage’ function of the deepTools2 suite.^82^

#### OK-seq analysis

OK-seq samples were analyzed as previously described.^84^ Briefly, processed OK-seq reads were aligned to the mm10 mouse genome with bowtie2 and stranded Watson and Crick read counts were binned into 1 kb bins. TSS/TTS genomic annotations for mm10 were obtained from the UCSC genome browser (https://genome.ucsc.edu/) and metagene tables were generated to include RNA-seq expression counts from naive control and EpiLC cell lines. Python was used to add 1 kb binned Watson and Crick reads from individual or combined OK-seq replicates to +/− 25 kb centered around metagene TSSs or region of interest-bed file peaks broken into quantiles using previously described Python scripts (https://github.com/FenyoLab/Ok-Seq_Processing).^84^ Strand bias data is presented as percent of forks moving left to right across TSSs by plotting the average Crick density and the strand reversed Watson density, or normalized Crick strand density calculated as Crick/(Watson+Crick). Statistics was performed by calculating the Crick density across 25 kb regions both upstream and downstream of the site of interest, and *p* values were calculated using a nonparametric Kruskal-Wallis H-test.

#### Genome track generation

Genome tracks for selected BigWig files were generated using trackplot.^85^ Chromosomal locations are specified in figure panels.

#### Metagene plots

Metagene plot data points were generated from BigWig files corresponding to each chromatin feature by using the ‘computeMatrix reference-point’ and ‘plotProfile’ functions of the deepTools2 package.^82^ Specifically, for Figure 4B the ‘computeMatrix reference-point’ function was run centered on 50kb genomic bins using the following settings: –binSize 5000 –upstream 250000 –downstream 250000 –averageTypeBins ‘mean’ –skipZeros –missingDataAsZero. For Figure 4C, plots were generated centered on H3K4me1 peaks^35^ using following settings: –binSize 250 –upstream 2500 –downstream 2500 –averageTypeBins ‘mean’ –skipZeros –missingDataAsZero. The ‘plotProfile’ function was used on the resulting.matrix file using the “–outFileNameData” to generate a tab separated file of data points constituting the metagene signal track. The signal data was then processed and plotted in R using the ‘geom_smooth’ function of the ‘ggplot2’ R package.

#### RT segmentation

Segmentation of RT from 50 kb RT bins was performed using circular binary segmentation.^30^ First, average WT naive RT and ΔRT (difference of EpiLC minus naive state RT) were separately used as input to the ‘segment’ parameter of the DNAcopy R package^30^ with the following settings: nperm = 1000, undo.splits = “sdundo”. Next, after iteratively optimizing undo.SD (the number of standard deviations between means to keep a split of segments) and alpha (significance level to accept a segment change point) parameters through visual inspection of plotted segments (Figure S2), final segmentation was performed using undo.SD = 5 and alpha = 1e-15. Resulting, naive and ΔRT segments were exported as bed files and used for machine learning analysis after incorporation of chromatin state data (see ‘Chromatin feature signal summation across RT segments’ section).

#### Chromatin feature signal summation across RT segments and IZ bins

The chromatin feature data were summarized for each RT or ΔRT segment using the following procedure. To obtain the background signal, reads corresponding to each individual chromatin feature in naive and EpiLC states were counted separately using featureCounts^86^ across the mm10 genome, with peak locations of the respective mark subtracted beforehand (obtained using bedtools subtract).^87^ The resulting feature-specific background signal from replicates was converted into counts per million (Cpm) and subtracted from the average across experimental replicates of each SEACR^81^ CUT&Tag consensus peak signal (see ‘CUT&Tag analysis’ section). Background-corrected peak signals for peaks overlapping more than 50% with an RT segment were then summed up for each segment. For ΔRT segments, the change in chromatin feature signal was calculated per segment by the difference of summed signal in EpiLC state and naive state. This resulted in a matrix with rows representing RT or ΔRT segments and columns representing naive RT or ΔRT and summed chromatin feature signal or its signal change for each of the 21 profiled features. This matrix was exported and served as input for machine learning (see ‘Machine learning of chromatin features predictive of RT’ section). For initiation zones (IZs) (Figure S5A), published IZs from naive ES cells^37^ were overlapped with 50 kb bins of the mm10 genome and bins we defined as IZ-positive or negative. Obtained classified bins were used as input for signal summation of naive CUT&Tag data as described above.

#### Integration of KMT2C/D loss-of-function epigenomics and RNA-seq with RT data

Published KMT2C^KO^, KMT2C/D^dKO^, KMT2C/D^dCD^ H3K4me1 CUT&RUN and RNA-seq data were re-analyzed as previously described.^35^ Briefly, for the union of each H3K4me1 SEACR consensus peak in naive and EpiLC states, the average Cpm read count for H3K4me1 from experimental replicates was calculated per genotype. The peaks were shown to be clearly enriched over IgG control.^35^ Next, the RNA-seq derived Cpm read count for the transcript corresponding to the transcriptional start site closest to each H3K4me1 peak was determined from the RNA-seq data. Then, log_2_ fold changes for H3K4me1 and RNA Cpm signal changes were calculated between genotypes and cellular state (EpiLC versus naive). Next, this data was integrated with the RT data (see ‘BioRepli-seq data analysis’ section) by calculating the median Cpm read count or log_2_ fold change for each 50kb genomic bin. Heatmaps were plotted using the ComplexHeatmap R package.^88^ Sankey plots were generated using the ggsankey R package.

#### Machine learning of chromatin features predictive of RT and IZ location

Matrices of background-corrected summed peak signal per RT segment or IZ bin (Figure S5A) (see section ‘Chromatin feature signal summation across RT segments and IZ bins’) were log-transformed (after addition of a pseudo-count of 0.01), normalized by genomic window size, and then used as input to the ‘train’ function of the caret R package to predict log_2_(RT), log_2_(ΔRT), or IZ presence (Figure S5A) using elastic net regression (“glmnet” method). Elastic Net regression is a regularized machine learning approach that penalizes the regression model for complexity while also keeping the model flexible enough to capture the underlying patterns in the data.^93^ Penalization shrinks less informative predictor variables (i.e., a certain chromatin feature that does not improve prediction of RT). The magnitude of non-zero parameter weights (coefficient assigned to each predictor variable) can be interpreted as the model’s way of highlighting which predictor variables have a more substantial impact on the outcome variable (RT, here). Each model underwent repeated cross validation through the use of the “repeatedcv” parameter in the ‘trControl’ function. This approach allows optimization of the model’s performance and helps counter overfitting of machine learning by partitioning the data into 5 random equally sized splits (using “fold” parameter) of which each iteratively serves as a test set while the rest is used as training set. This process was repeated 5 times to obtain the optimal model (using “repeats” parameter). Centering and scaling pre-processing was specified in the ‘train’ function to standardize the range of the continuous variables before applying elastic net regression, ensuring that coefficients are estimated on the same scale and regularized appropriately. Predicted values were extracted using the ‘extractPrediction’ function and the dimensionless parameter weights for all predictor variables were extracted using the ‘coef’ function on the final model. Spearman correlations were computed using the ‘cor’ function with the setting “method = spearman”.

#### Real-time quantitative RT PCR

RNA was extracted using TRIzol according to manufacturers’ instructions. RNA was reverse transcribed using Maxima First Strand cDNA Synthesis Kit and cDNA amplified using target-specific primers in 1x SYBR Green master mix. RNA levels were determined using the ΔΔCt method. Undetected measurements were imputed at 45 cycles.

### QUANTIFICATION AND STATISTICAL ANALYSIS

Statistical tests were performed in R. Detailed information about test type, error bars and sample sizes are included in all figures and their legends with further specification in the STAR Methods section. R scripts used for all analysis were deposited at https://github.com/dgoekbuget. No samples or data were excluded from the analysis.

## Supplementary Material

1

## Figures and Tables

**Table:** KEY RESOURCES TABLE

REAGENT or RESOURCE	SOURCE	IDENTIFIER
Antibodies		

Rabbit IgG	Abcam	Cat# ab171870; RRID:AB_2687657
H3K4me1	Abcam	Cat# ab8895; RRID:AB_306847
H3K4me2	Abcam	Cat# ab7766; RRID:AB_2560996
H3K4me3	Abcam	Cat# ab8580; RRID:AB_306649
H3K27ac	Abcam	Cat# ab4729; RRID:AB_2118291
H3K27me3	Cell Signaling	Cat# 9733; RRID:AB_2616029
H3K36me1	Cell Signaling	Cat# 14111; RRID:AB_2798395
H3K36me2	Cell Signaling	Cat# 2901; RRID:AB_1030983
H2BK5ac	Cell Signaling	Cat# 12799; RRID:AB_2636805
H3K9ac	Cell Signaling	Cat# 9649; RRID:AB_823528
H3K9me3	Active Motif	Cat# 39162; RRID:AB_2532132
H4K20me1	Diagenode	Cat# MAb-147-100; RRID: N/A
RAD21	GeneTex	Cat# GTX106012; RRID:AB_11176384
RNApol-II-S2/5P	Cell Signaling	Cat# 13546; RRID:AB_2798253
H4K8ac	Cell Signaling	Cat# 2594; RRID:AB_2248400
H4K16ac	Millipore	Cat# 07-329; RRID:AB_310525
H3K14ac	Cell Signaling	Cat# 7627; RRID:AB_10839410
H3K9me2	Active Motif	Cat# 39240; RRID:AB_2793199
H3.3	Active Motif	Cat# 91192; RRID:AB_2793796
H2A.Z	Active Motif	Cat# 39944; RRID:AB_2793401
H2AK119ub	Cell Signaling	Cat# 8240; RRID:AB_10891618
H2AX-S139P	Cell Signaling	Cat# 2577; RRID:AB_2118010
Guinea pig anti-rabbit	Antibodies Online	Cat# ABIN101961; RRID:AB_10775589

Chemicals, peptides, and recombinant proteins		

KO DMEM	ThermoFisher Scientific	10829018
Fetal bovine serum	Corning	35-010-CV
NEAA	ThermoFisher Scientific	11140035
L-Glutamine	ThermoFisher Scientific	59202C-100ML
Penicillin/Streptomycin	Millipore Sigma	P4333-100ML
PD0325901	Axon Medchem	1408
CHIR99021	Axon Medchem	1386
4-Hydroxytamoxifen	Millipore Sigma	5082250001
LIF	In-house	N/A
1x PBS	ThermoFisher Scientific	10010023
EdU	ThermoFisher Scientific	A10044
Trypsin-EDTA	ThermoFisher Scientific	25200072
35mm cell strainer	Corning	352235
Ethanol	Koptec	V1016
PI/RNase	ThermoFisher Scientific	501121519
10x TBS	ThermoFisher Scientific	J62938.K7
Biotin Azide	ThermoFisher Scientific	B10184
CUSO4	Millipore Sigma	C1297-100G
THPTA	Millipore Sigma	762342-100MG
Na-Ascorbate	VWR	95035-692
Tris-HCl pH 7.5	Teknova	T5110
EDTA pH 8	ThermoFisher Scientific	AM9260G
NaCI	ThermoFisher Scientific	AM9760G
Tween 20	Millipore Sigma	P9416-50ML
TE pH 8	ThermoFisher Scientific	No.AM9849
HEPES buffer pH 7.5	Millipore Sigma	H3375
Spermidine	Millipore Sigma	S2501
Protease inhibitors	Millipore Sigma	11836170001
Digitonin	Millipore Sigma	300410
pA-Tn5	QB3 Macrolabs (UC Berkeley)	N/A
MgCl2	Millipore Sigma	M8266-100G
Proteinase K	ThermoFisher Scientific	E00492
SDS	ThermoFisher Scientific	AM9822
Biotin TEG Azide	Berry & Associates, Inc.	BA0038
NaOH	Millipore Sigma	71689
TRIzol	ThermoFisher Scientific	15596026

Critical commercial assays		

NucleoSpin Tissue kit	Macherey-Nagel	740952.50
Streptavidin beads	NEB	S1420S
NEBNext Ultra II	NEB	E7645S
Agencourt Ampure XP beads	Beckman Coulter	A63880
SPRIselect Reagent	Beckman Coulter	B23317
Concanavalin A beads	Bang Laboratories	BP531
Tapestation D1000 tapes	Agilent	UFUC0P-5067-5582
Dynabeads MyOne Streptavidin T1	ThermoFisher Scientific	65601
Maxima First Strand cDNA Synthesis Kit for RT-qPCR	ThermoFisher Scientific	K1642
SYBR Green Master Mix for qPCR	ThermoFisher Scientific	A46012

Deposited data		

H3K4me1 C&T	This study	GEO: GSE216475
H3K4me2 C&T	This study	GEO: GSE216475
H3K4me3 C&T	This study	GEO: GSE216475
H3K27me3 C&T	This study	GEO: GSE216475
H3K9me2 C&T	This study	GEO: GSE216475
H3K9me3 C&T	This study	GE0: GSE216475
H4K20me1 C&T	This study	GEO: GSE216475
H3K36me1 C&T	This study	GEO: GSE216475
H3K36me2 C&T	This study	GEO: GSE216475
H2BK5ac C&T	This study	GEO: GSE216475
H3K9ac C&T	This study	GEO: GSE216475
H3K14ac C&T	This study	GEO: GSE216475
H3K27ac C&T	This study	GEO: GSE216475
H4K8ac C&T	This study	GEO: GSE216475
H4K16ac C&T	This study	GEO: GSE216475
P-Pol II C&T	This study	GEO: GSE216475
H2A.Z C&T	This study	GEO: GSE216475
H3.3 C&T	This study	GEO: GSE216475
gH2AX C&T	This study	GEO: GSE216475
RAD21 C&T	This study	GEO: GSE216475
H2BK119Ub C&T	This study	GEO: GSE216475
H3K4me1 C&R	Boileau et al. 2023^35^	GEO: GSE212950
OK-seq (Naive WT)	Petryk et al. 2018^37^	GEO: GSE117274
OK-seq	This study	GEO: GSE216475
NAIL-seq	Liu et al. 2021^17^	GEO: GSE174680
SNS-seq	Cayrou et al. 2015^41^	GEO: GSE68347
BrdU-Repli-seq	Miuraetal. 2019^29^	GEO: GSE113985
Bio-Repli-seq	This study	GEO: GSE216475
RNA-seq	Boileau et al. 2023^35^	GEO: GSE212950

Experimental models: Cell lines		

v6.5 mouse ES cells	Novus Biologicals	NBP1-41162
KMT2C^KO^/KMT2D^cKO^ mouse ES cells^56^	Kindly provided by Dr. Kai Ge (NIH)	N/A
KMT2C/D^dCD^ mouse ES cells^36^	Kindly provided by Dr. Joanna Wysocka (Stanford)	N/A

Oligonucleotides		

Kmt2d-KO-qPCR-F: AGGACATGTGTGTGGTGTG	IDT	N/A
Kmt2d-KO-qPCR-R: ACCTTGGTGATCTTGCTGTT	IDT	N/A
Gapdh-qPCR-F: GACTTCAACAGCAACTCCCAC	IDT	N/A
Gapdh-qPCR-R: TCCACCACCCTGTTGCTGTA	IDT	N/A

Software and algorithms		

bowtie2	Langmead et al. 2012^79^	https://github.com/BenLangmead/bowtie2
rstudio server	Posit	https://posit.co/download/rstudio-server/
nfcore/cutandrun	Ewels et al. 2020^80^	https://nf-co.re/cutandrun/
SEACR	Meers et al. 2019^81^	https://github.com/FredHutch/SEACR
deepTools2	Ramirez et al. 2016^82^	https://deeptools.readthedocs.io/en/develop/
UCSC genome browser	Perez et al. 2024^83^	https://genome.ucsc.edu/
OK-seq processing scripts	Kit Leng Lui et al. 2021^84^	https://github.com/FenyoLab/Ok-Seq_Processing
trackplot	Pohl and Beato 2014^85^	https://github.com/PoisonAlien/trackplot
ggplot2	Wickham et al.	https://ggplot2.tidyverse.org
DNAcopy	Olshen et al. 2004^30^	https://bioconductor.org/packages/release/bioc/html/DNAcopy.html
featureCounts	Liao et al. 2014^86^	https://subread.sourceforge.net/featureCounts.html
bedtools2	Quinlan and Hall 2010^87^	https://bedtools.readthedocs.io/en/latest/
caret	Kuhn et al.	https://cran.r-project.org/web/packages/caret/index.html
ComplexHeatmap	Guetal. 2016^88^	https://bioconductor.org/packages/release/bioc/html/ComplexHeatmap.html
ggsankey	Sjoberg	https://github.com/davidsjoberg/ggsankey
Data processing and analysis	This paper	https://github.com/dgoekbuget

## Highlights

- Chromatin state predicts local replication timing changes during ESC differentiation
- H3K4me1 emerges as a top predictor of replication timing change
- Loss of H3K4me1 locally impairs replication timing changes independent of transcription
- H3K4me1 locally promotes replication origin activation

## References

[1] Marchal C, Sima J, and Gilbert DM (2019). Control of DNA replication timing in the 3D genome. Nat. Rev. Mol. Cell Biol 20, 721–737. 10.1038/s41580-019-0162-y. 31477886 PMC11567694

[2] Ryba T, Hiratani I, Lu J, Itoh M, Kulik M, Zhang J, Schulz TC, Robins AJ, Dalton S, and Gilbert DM (2010). Evolutionarily conserved replication timing profiles predict long-range chromatin interactions and distinguish closely related cell types. Genome Res. 20, 761–770. 10.1101/gr.099655.109. 20430782 PMC2877573

[3] Hiratani I, Ryba T, Itoh M, Yokochi T, Schwaiger M, Chang C-W, Lyou Y, Townes TM, Schübeler D, and Gilbert DM (2008). Global reorganization of replication domains during embryonic stem cell differentiation. PLoS Biol. 6, e245. 10.1371/journal.pbio.0060245. 18842067 PMC2561079

[4] Hiratani I, Ryba T, Itoh M, Rathjen J, Kulik M, Papp B, Fussner E, Bazett-Jones DP, Plath K, Dalton S, (2010). Genome-wide dynamics of replication timing revealed by in vitro models of mouse embryogenesis. Genome Res. 20, 155–169. 10.1101/gr.099796.109. 19952138 PMC2813472

[5] MacAlpine DM, Rodríguez HK, and Bell SP (2004). Coordination of replication and transcription along a Drosophila chromosome. Genes Dev. 18, 3094–3105. 10.1101/gad.1246404. 15601823 PMC535919

[6] Schübeler D, Scalzo D, Kooperberg C, van Steensel B, Delrow J, and Groudine M. (2002). Genome-wide DNA replication profile for Drosophila melanogaster: a link between transcription and replication timing. Nat. Genet 32, 438–442. 10.1038/ng1005. 12355067

[7] Woodfine K, Fiegler H, Beare DM, Collins JE, McCann OT, Young BD, Debernardi S, Mott R, Dunham I, and Carter NP (2004). Replication timing of the human genome. Hum. Mol. Genet 13, 191–202. 10.1093/hmg/ddh016. 14645202

[8] White EJ, Emanuelsson O, Scalzo D, Royce T, Kosak S, Oakeley EJ, Weissman S, Gerstein M, Groudine M, Snyder M, and Schübeler D. (2004). DNA replication-timing analysis of human chromosome 22 at high resolution and different developmental states. Proc. Natl. Acad. Sci. USA 101, 17771–17776. 10.1073/pnas.0408170101. 15591350 PMC539744

[9] Goldman MA, Holmquist GP, Gray MC, Caston LA, and Nag A. (1984). Replication timing of genes and middle repetitive sequences. Science 224, 686–692. 10.1126/science.6719109. 6719109

[10] Yue F, Cheng Y, Breschi A, Vierstra J, Wu W, Ryba T, Sandstrom R, Ma Z, Davis C, Pope BD, (2014). A comparative encyclopedia of DNA elements in the mouse genome. Nature 515, 355–364. 10.1038/nature13992. 25409824 PMC4266106

[11] Yaffe E, Farkash-Amar S, Polten A, Yakhini Z, Tanay A, and Simon I. (2010). Comparative analysis of DNA replication timing reveals conserved large-scale chromosomal architecture. PLoS Genet. 6, e1001011. 10.1371/journal.pgen.1001011. 20617169 PMC2895651

[12] Rao SSP, Huntley MH, Durand NC, Stamenova EK, Bochkov ID, Robinson JT, Sanborn AL, Machol I, Omer AD, Lander ES, and Aiden EL (2014). A 3D map of the human genome at kilobase resolution reveals principles of chromatin looping. Cell 159, 1665–1680. 10.1016/j.cell.2014.11.021. 25497547 PMC5635824

[13] Emerson DJ, Zhao PA, Cook AL, Barnett RJ, Klein KN, Saulebekova D, Ge C, Zhou L, Simandi Z, Minsk MK, (2022). Cohesin-mediated loop anchors confine the locations of human replication origins. Nature 606, 812–819. 10.1038/s41586-022-04803-0. 35676475 PMC9217744

[14] Pope BD, Ryba T, Dileep V, Yue F, Wu W, Denas O, Vera DL, Wang Y, Hansen RS, Canfield TK, (2014). Topologically associating domains are stable units of replication-timing regulation. Nature 515, 402–405. 10.1038/nature13986. 25409831 PMC4251741

[15] Moindrot B, Audit B, Klous P, Baker A, Thermes C, de Laat W, Bouvet P, Mongelard F, and Arneodo A. (2012). 3D chromatin conformation correlates with replication timing and is conserved in resting cells. Nucleic Acids Res. 40, 9470–9481. 10.1093/nar/gks736. 22879376 PMC3479194

[16] Sima J, Chakraborty A, Dileep V, Michalski M, Klein KN, Holcomb NP, Turner JL, Paulsen MT, Rivera-Mulia JC, Trevilla-Garcia C, (2019). Identifying cis elements for spatiotemporal control of mammalian DNA replication. Cell 176, 816–830.e18. 10.1016/j.cell.2018.11.036. 30595451 PMC6546437

[17] Liu Y, Ai C, Gan T, Wu J, Jiang Y, Liu X, Lu R, Gao N, Li Q, Ji X, and Hu J. (2021). Transcription shapes DNA replication initiation to preserve genome integrity. Genome Biol. 22, 176. 10.1186/s13059-021-02390-3. 34108027 PMC8188667

[18] O’Keefe RT, Henderson SC, and Spector DL (1992). Dynamic organization of DNA replication in mammalian cell nuclei: spatially and temporally defined replication of chromosome-specific alpha-satellite DNA sequences. J. Cell Biol 116, 1095–1110. 10.1083/jcb.116.5.1095. 1740468 PMC2289349

[19] Visser AE, Eils R, Jauch A, Little G, Bakker PJ, Cremer T, and Aten JA (1998). Spatial distributions of early and late replicating chromatin in interphase chromosome territories. Exp. Cell Res 243, 398–407. 10.1006/excr.1998.4144. 9743599

[20] Bass HW, Hoffman GG, Lee T-J, Wear EE, Joseph SR, Allen GC, Hanley-Bowdoin L, and Thompson WF (2015). Defining multiple, distinct, and shared spatiotemporal patterns of DNA replication and endoreduplication from 3D image analysis of developing maize (Zea mays L.) root tip nuclei. Plant Mol. Biol 89, 339–351. 10.1007/s11103-015-0364-4. 26394866 PMC4631726

[21] Akhtar A, and Gasser SM (2007). The nuclear envelope and transcriptional control. Nat. Rev. Genet 8, 507–517. 10.1038/nrg2122. 17549064

[22] Schübeler D, Francastel C, Cimbora DM, Reik A, Martin DI, and Groudine M. (2000). Nuclear localization and histone acetylation: a pathway for chromatin opening and transcriptional activation of the human beta-globin locus. Genes Dev. 14, 940–950. 10783166 PMC316536

[23] Goren A, Tabib A, Hecht M, and Cedar H. (2008). DNA replication timing of the human beta-globin domain is controlled by histone modification at the origin. Genes Dev. 22, 1319–1324. 10.1101/gad.468308. 18443145 PMC2377185

[24] Krishnakumar R, Chen AF, Pantovich MG, Danial M, Parchem RJ, Labosky PA, and Blelloch R. (2016). FOXD3 regulates pluripotent stem cell potential by simultaneously initiating and repressing enhancer activity. Cell Stem Cell 18, 104–117. 10.1016/j.stem.2015.10.003. 26748757 PMC4775235

[25] Chen AF, Liu AJ, Krishnakumar R, Freimer JW, DeVeale B, and Blelloch R. (2018). GRHL2-Dependent Enhancer Switching Maintains a Pluripotent Stem Cell Transcriptional Subnetwork after Exit from Naive Pluripotency. Cell Stem Cell 23, 226–238.e4. 10.1016/j.stem.2018.06.005. 30017589 PMC6456389

[26] Parchem RJ, Ye J, Judson RL, LaRussa MF, Krishnakumar R, Blelloch A, Oldham MC, and Blelloch R. (2014). Two miRNA clusters reveal alternative paths in late-stage reprogramming. Cell Stem Cell 14, 617–631. 10.1016/j.stem.2014.01.021. 24630794 PMC4305531

[27] Yang P, Humphrey SJ, Cinghu S, Pathania R, Oldfield AJ, Kumar D, Perera D, Yang JYH, James DE, Mann M, and Jothi R. (2019). Multi-omic Profiling Reveals Dynamics of the Phased Progression of Pluripotency. Cell Syst. 8, 427–445.e10. 10.1016/j.cels.2019.03.012. 31078527 PMC6544180

[28] Smith A. (2017). Formative pluripotency: the executive phase in a developmental continuum. Development 144, 365–373. 10.1242/dev.142679. 28143843 PMC5430734

[29] Miura H, Takahashi S, Poonperm R, Tanigawa A, Takebayashi S-I, and Hiratani I. (2019). Single-cell DNA replication profiling identifies spatiotemporal developmental dynamics of chromosome organization. Nat. Genet 51, 1356–1368. 10.1038/s41588-019-0474-z. 31406346

[30] Olshen AB, Venkatraman ES, Lucito R, and Wigler M. (2004). Circular binary segmentation for the analysis of array-based DNA copy number data. Biostatistics 5, 557–572. 10.1093/biostatistics/kxh008. 15475419

[31] Calo E, and Wysocka J. (2013). Modification of enhancer chromatin: what, how, and why? Mol. Cell 49, 825–837. 10.1016/j.molcel.2013.01.038. 23473601 PMC3857148

[32] Herz H-M, Mohan M, Garruss AS, Liang K, Takahashi Y-H, Mickey K, Voets O, Verrijzer CP, and Shilatifard A. (2012). Enhancer-associated H3K4 monomethylation by Trithorax-related, the Drosophila homolog of mammalian Mll3/Mll4. Genes Dev. 26, 2604–2620. 10.1101/gad.201327.112. 23166019 PMC3521626

[33] Hu D, Gao X, Morgan MA, Herz H-M, Smith ER, and Shilatifard A. (2013). The MLL3/MLL4 branches of the COMPASS family function as major histone H3K4 monomethylases at enhancers. Mol. Cell Biol 33, 4745–4754. 10.1128/MCB.01181-13. 24081332 PMC3838007

[34] Cao K, Collings CK, Marshall SA, Morgan MA, Rendleman EJ, Wang L, Sze CC, Sun T, Bartom ET, and Shilatifard A. (2017). SET1A/COMPASS and shadow enhancers in the regulation of homeotic gene expression. Genes Dev. 31, 787–801. 10.1101/gad.294744.116. 28487406 PMC5435891

[35] Boileau RM, Chen KX, and Blelloch R. (2023). Loss of MLL3/4 decouples enhancer H3K4 monomethylation, H3K27 acetylation, and gene activation during embryonic stem cell differentiation. Genome Biol. 24, 41. 10.1186/s13059-023-02883-3. 36869380 PMC9983171

[36] Dorighi KM, Swigut T, Henriques T, Bhanu NV, Scruggs BS, Nady N, Still CD, Garcia BA, Adelman K, and Wysocka J. (2017). Mll3 and Mll4 Facilitate Enhancer RNA Synthesis and Transcription from Promoters Independently of H3K4 Monomethylation. Mol. Cell 66, 568–576.e4. 10.1016/j.molcel.2017.04.018. 28483418 PMC5662137

[37] Petryk N, Dalby M, Wenger A, Stromme CB, Strandsby A, Andersson R, and Groth A. (2018). MCM2 promotes symmetric inheritance of modified histones during DNA replication. Science 361, 1389–1392. 10.1126/science.aau0294. 30115746

[38] Chen Y-H, Keegan S, Kahli M, Tonzi P, Fenyö D, Huang TT, and Smith DJ (2019). Transcription shapes DNA replication initiation and termination in human cells. Nat. Struct. Mol. Biol 26, 67–77. 10.1038/s41594-018-0171-0. 30598550 PMC6320713

[39] Prioleau M-N, and MacAlpine DM (2016). DNA replication origins-where do we begin? Genes Dev. 30, 1683–1697. 10.1101/gad.285114.116. 27542827 PMC5002974

[40] Petryk N, Kahli M, d’Aubenton-Carafa Y, Jaszczyszyn Y, Shen Y, Silvain M, Thermes C, Chen C-L, and Hyrien O. (2016). Replication landscape of the human genome. Nat. Commun 7, 10208. 10.1038/ncomms10208. 26751768 PMC4729899

[41] Cayrou C, Ballester B, Peiffer I, Fenouil R, Coulombe P, Andrau J-C, van Helden J, and Méchali M. (2015). The chromatin environment shapes DNA replication origin organization and defines origin classes. Genome Res. 25, 1873–1885. 10.1101/gr.192799.115. 26560631 PMC4665008

[42] Yu W, Zhong Q, Wen Z, Zhang W, and Huang Y. (2022). Genome architecture plasticity underlies DNA replication timing dynamics in cell differentiation. Front. Genet 13, 961612. 10.3389/fgene.2022.961612. 36118849 PMC9478753

[43] Dileep V, Wilson KA, Marchal C, Lyu X, Zhao PA, Li B, Poulet A, Bartlett DA, Rivera-Mulia JC, Qin ZS, (2019). Rapid Irreversible Transcriptional Reprogramming in Human Stem Cells Accompanied by Discordance between Replication Timing and Chromatin Compartment. Stem Cell Rep. 13, 193–206. 10.1016/j.stemcr.2019.05.021. PMC6627004 31231024

[44] Eaton ML, Prinz JA, MacAlpine HK, Tretyakov G, Kharchenko PV, and MacAlpine DM (2011). Chromatin signatures of the Drosophila replication program. Genome Res. 21, 164–174. 10.1101/gr.116038.110. 21177973 PMC3032920

[45] Gatto A, Forest A, Quivy J-P, and Almouzni G. (2022). HIRA-dependent boundaries between H3 variants shape early replication in mammals. Mol. Cell 82, 1909–1923.e5. 10.1016/j.molcel.2022.03.017. 35381196

[46] Meeks JJ, and Shilatifard A. (2017). Multiple roles for the MLL/COMPASS family in the epigenetic regulation of gene expression and in cancer. Annu. Rev. Cancer Biol 1, 425–446. 10.1146/annurev-cancerbio-050216-034333.

[47] Ashokkumar D, Zhang Q, Much C, Bledau AS, Naumann R, Alexopoulou D, Dahl A, Goveas N, Fu J, Anastassiadis K, (2020). MLL4 is required after implantation, whereas MLL3 becomes essential during late gestation. Development 147, dev186999. 10.1242/dev.186999. 32439762

[48] Bledau AS, Schmidt K, Neumann K, Hill U, Ciotta G, Gupta A, Torres DC, Fu J, Kranz A, Stewart AF, and Anastassiadis K. (2014). The H3K4 methyltransferase Setd1a is first required at the epiblast stage, whereas Setd1b becomes essential after gastrulation. Development 141, 1022–1035. 10.1242/dev.098152. 24550110

[49] Glaser S, Schaft J, Lubitz S, Vintersten K, van der Hoeven F, Tufteland KR, Aasland R, Anastassiadis K, Ang S-L, and Stewart AF (2006). Multiple epigenetic maintenance factors implicated by the loss of Mll2 in mouse development. Development 133, 1423–1432. 10.1242/dev.02302. 16540515

[50] Xie G, Lee J-E, Senft AD, Park Y-K, Jang Y, Chakraborty S, Thompson JJ, McKernan K, Liu C, Macfarlan TS, (2023). MLL3/MLL4 methyltransferase activities control early embryonic development and embryonic stem cell differentiation in a lineage-selective manner. Nat. Genet 55, 693–705. 10.1038/s41588-023-01356-4. 37012455 PMC12269479

[51] Miller T, Krogan NJ, Dover J, Erdjument-Bromage H, Tempst P, Johnston M, Greenblatt JF, and Shilatifard A. (2001). COMPASS: a complex of proteins associated with a trithorax-related SET domain protein. Proc. Natl. Acad. Sci. USA 98, 12902–12907. 10.1073/pnas.231473398. 11687631 PMC60797

[52] Nislow C, Ray E, and Pillus L. (1997). SET1, a yeast member of the trithorax family, functions in transcriptional silencing and diverse cellular processes. Mol. Biol. Cell 8, 2421–2436. 10.1091/mbc.8.12.2421. 9398665 PMC25717

[53] Rickels R, Herz H-M, Sze CC, Cao K, Morgan MA, Collings CK, Gause M, Takahashi Y-H, Wang L, Rendleman EJ, (2017). Histone H3K4 monomethylation catalyzed by Trr and mammalian COMPASS-like proteins at enhancers is dispensable for development and viability. Nat. Genet 49, 1647–1653. 10.1038/ng.3965. 28967912 PMC5663216

[54] Wu J, Liu Y, Zhangding Z, Liu X, Ai C, Gan T, Liang H, Guo Y, Chen M, Liu Y, (2023). Cohesin maintains replication timing to suppress DNA damage on cancer genes. Nat. Genet 55, 1347–1358. 10.1038/s41588-023-01458-z. 37500731

[55] Lee J-E, Wang C, Xu S, Cho Y-W, Wang L, Feng X, Baldridge A, Sartorelli V, Zhuang L, Peng W, and Ge K. (2013). H3K4 mono- and dimethyltransferase MLL4 is required for enhancer activation during cell differentiation. Elife 2, e01503. 10.7554/eLife.01503. 24368734 PMC3869375

[56] Wang C, Lee J-E, Lai B, Macfarlan TS, Xu S, Zhuang L, Liu C, Peng W, and Ge K. (2016). Enhancer priming by H3K4 methyltransferase MLL4 controls cell fate transition. Proc. Natl. Acad. Sci. USA 113, 11871–11876. 10.1073/pnas.1606857113. 27698142 PMC5081576

[57] Fulco CP, Nasser J, Jones TR, Munson G, Bergman DT, Subramanian V, Grossman SR, Anyoha R, Doughty BR, Patwardhan TA, (2019). Activity-by-contact model of enhancer-promoter regulation from thousands of CRISPR perturbations. Nat. Genet 51, 1664–1669. 10.1038/s41588-019-0538-0. 31784727 PMC6886585

[58] Nasser J, Bergman DT, Fulco CP, Guckelberger P, Doughty BR, Patwardhan TA, Jones TR, Nguyen TH, Ulirsch JC, Lekschas F, (2021). Genome-wide enhancer maps link risk variants to disease genes. Nature 593, 238–243. 10.1038/s41586-021-03446-x. 33828297 PMC9153265

[59] Cornacchia D, Dileep V, Quivy J-P, Foti R, Tili F, Santarella-Mellwig R, Antony C, Almouzni G, Gilbert DM, and Buonomo SBC (2012). Mouse Rif1 is a key regulator of the replication-timing programme in mammalian cells. EMBO J. 31, 3678–3690. 10.1038/emboj.2012.214. 22850673 PMC3442270

[60] Yamazaki S, Ishii A, Kanoh Y, Oda M, Nishito Y, and Masai H. (2012). Rif1 regulates the replication timing domains on the human genome. EMBO J. 31, 3667–3677. 10.1038/emboj.2012.180. 22850674 PMC3442267

[61] Foti R, Gnan S, Cornacchia D, Dileep V, Bulut-Karslioglu A, Diehl S, Buness A, Klein FA, Huber W, Johnstone E, (2016). Nuclear Architecture Organized by Rif1 Underpins the Replication-Timing Program. Mol. Cell 61, 260–273. 10.1016/j.molcel.2015.12.001. 26725008 PMC4724237

[62] Klein KN, Zhao PA, Lyu X, Sasaki T, Bartlett DA, Singh AM, Tasan I, Zhang M, Watts LP, Hiraga S-I, (2021). Replication timing maintains the global epigenetic state in human cells. Science 372, 371–378. 10.1126/science.aba5545. 33888635 PMC8173839

[63] Kirstein N, Buschle A, Wu X, Krebs S, Blum H, Kremmer E, Vorberg IM, Hammerschmidt W, Lacroix L, Hyrien O, (2021). Human ORC/MCM density is low in active genes and correlates with replication time but does not delimit initiation zones. Elife 10, e62161. 10.7554/eLife.62161. 33683199 PMC7993996

[64] Local A, Huang H, Albuquerque CP, Singh N, Lee AY, Wang W, Wang C, Hsia JE, Shiau AK, Ge K, (2018). Identification of H3K4me1-associated proteins at mammalian enhancers. Nat. Genet 50, 73–82. 10.1038/s41588-017-0015-6. 29255264 PMC6007000

[65] Dungrawala H, Rose KL, Bhat KP, Mohni KN, Glick GG, Couch FB, and Cortez D. (2015). The Replication Checkpoint Prevents Two Types of Fork Collapse without Regulating Replisome Stability. Mol. Cell 59, 998–1010. 10.1016/j.molcel.2015.07.030. 26365379 PMC4575883

[66] Sparvoli E, Levi M, and Rossi E. (1994). Replicon clusters may form structurally stable complexes of chromatin and chromosomes. J. Cell Sci 107, 3097–3103. 10.1242/jcs.107.11.3097. 7699008

[67] Ferreira J, Paolella G, Ramos C, and Lamond AI (1997). Spatial organization of large-scale chromatin domains in the nucleus: a magnified view of single chromosome territories. J. Cell Biol 139, 1597–1610. 10.1083/jcb.139.7.1597. 9412456 PMC2132633

[68] Yan J, Chen S-AA, Local A, Liu T, Qiu Y, Dorighi KM, Preissl S, Rivera CM, Wang C, Ye Z, (2018). Histone H3 lysine 4 monomethylation modulates long-range chromatin interactions at enhancers. Cell Res. 28, 204–220. 10.1038/cr.2018.1. 29313530 PMC5799818

[69] Behera V, Stonestrom AJ, Hamagami N, Hsiung CC, Keller CA, Giardine B, Sidoli S, Yuan Z-F, Bhanu NV, Werner MT, (2019). Interrogating histone acetylation and BRD4 as mitotic bookmarks of transcription. Cell Rep. 27, 400–415.e5. 10.1016/j.celrep.2019.03.057. 30970245 PMC6664437

[70] Kang H, Shokhirev MN, Xu Z, Chandran S, Dixon JR, and Hetzer MW (2020). Dynamic regulation of histone modifications and long-range chromosomal interactions during postmitotic transcriptional reactivation. Genes Dev. 34, 913–930. 10.1101/gad.335794.119. 32499403 PMC7328517

[71] Blobel GA, Kadauke S, Wang E, Lau AW, Zuber J, Chou MM, and Vakoc CR (2009). A reconfigured pattern of MLL occupancy within mitotic chromatin promotes rapid transcriptional reactivation following mitotic exit. Mol. Cell 36, 970–983. 10.1016/j.molcel.2009.12.001. 20064463 PMC2818742

[72] Peycheva M, Neumann T, Malzl D, Nazarova M, Schoeberl UE, and Pavri R. (2022). DNA replication timing directly regulates the frequency of oncogenic chromosomal translocations. Science 377, eabj5502. 10.1126/science.abj5502. 36108018

[73] Xu S, Wang N, Zuccaro MV, Gerhardt J, Iyyappan R, Scatolin GN, Jiang Z, Baslan T, Koren A, and Egli D. (2024). DNA replication in early mammalian embryos is patterned, predisposing lamina-associated regions to fragility. Nat. Commun 15, 5247. 10.1038/s41467-024-49565-7. 38898078 PMC11187207

[74] Du Z, Zheng H, Huang B, Ma R, Wu J, Zhang X, He J, Xiang Y, Wang Q, Li Y, (2017). Allelic reprogramming of 3D chromatin architecture during early mammalian development. Nature 547, 232–235. 10.1038/nature23263. 28703188

[75] Flyamer IM, Gassler J, Imakaev M, Brandão HB, Ulianov SV, Abdennur N, Razin SV, Mirny LA, and Tachibana-Konwalski K. (2017). Single-nucleus Hi-C reveals unique chromatin reorganization at oocyte-to-zygote transition. Nature 544, 110–114. 10.1038/nature21711. 28355183 PMC5639698

[76] Ke Y, Xu Y, Chen X, Feng S, Liu Z, Sun Y, Yao X, Li F, Zhu W, Gao L, (2017). 3D Chromatin Structures of Mature Gametes and Structural Reprogramming during Mammalian Embryogenesis. Cell 170, 367–381.e20. 10.1016/j.cell.2017.06.029. 28709003

[77] Mendiratta G, Ke E, Aziz M, Liarakos D, Tong M, and Stites EC (2021). Cancer gene mutation frequencies for the U.S. population. Nat. Commun 12, 5961. 10.1038/s41467-021-26213-y. 34645806 PMC8514428

[78] Long H, Zhang L, Lv M, Wen Z, Zhang W, Chen X, Zhang P, Li T, Chang L, Jin C, (2020). H2A.Z facilitates licensing and activation of early replication origins. Nature 577, 576–581. 10.1038/s41586-019-1877-9. 31875854

[79] Langmead B, and Salzberg SL (2012). Fast gapped-read alignment with Bowtie 2. Nat. Methods 9, 357–359. 10.1038/nmeth.1923. 22388286 PMC3322381

[80] Ewels PA, Peltzer A, Fillinger S, Patel H, Alneberg J, Wilm A, Garcia MU, Di Tommaso P, and Nahnsen S. (2020). The nf-core framework for community-curated bioinformatics pipelines. Nat. Biotechnol 38, 276–278. 10.1038/s41587-020-0439-x. 32055031

[81] Meers MP, Tenenbaum D, and Henikoff S. (2019). Peak calling by Sparse Enrichment Analysis for CUTnRUN chromatin profiling. Epigenet. Chromatin 12, 42. 10.1186/s13072-019-0287-4. PMC6624997 31300027

[82] Ramírez F, Ryan DP, Grüning B, Bhardwaj V, Kilpert F, Richter AS, Heyne S, Dündar F, and Manke T. (2016). deepTools2: a next generation web server for deep-sequencing data analysis. Nucleic Acids Res. 44, W160–W165. 10.1093/nar/gkw257. 27079975 PMC4987876

[83] Perez G, Barber GP, Benet-Pages A, Casper J, Clawson H, Diekhans M, Fischer C, Gonzalez JN, Hinrichs AS, Lee CM, (2025). The UCSC Genome Browser database: 2025 update. Nucleic Acids Res. 53, D1243–D1249. 10.1093/nar/gkae974. 39460617 PMC11701590

[84] Kit Leng Lui S, Keegan S, Tonzi P, Kahli M, Chen Y-H, Chalhoub N, Coleman KE, Fenyo D, Smith DJ, and Huang TT (2021). Monitoring genome-wide replication fork directionality by Okazaki fragment sequencing in mammalian cells. Nat. Protoc 16, 1193–1218. 10.1038/s41596-020-00454-5. 33442052 PMC8792808

[85] Pohl A, and Beato M. (2014). bwtool: a tool for bigWig files. Bioinformatics 30, 1618–1619. 10.1093/bioinformatics/btu056. 24489365 PMC4029031

[86] Liao Y, Smyth GK, and Shi W. (2014). featureCounts: an efficient general purpose program for assigning sequence reads to genomic features. Bioinformatics 30, 923–930. 10.1093/bioinformatics/btt656. 24227677

[87] Quinlan AR, and Hall IM (2010). BEDTools: a flexible suite of utilities for comparing genomic features. Bioinformatics 26, 841–842. 10.1093/bioinformatics/btq033. 20110278 PMC2832824

[88] Gu Z, Eils R, and Schlesner M. (2016). Complex heatmaps reveal patterns and correlations in multidimensional genomic data. Bioinformatics 32, 2847–2849. 10.1093/bioinformatics/btw313. 27207943

[89] Marchal C, Sasaki T, Vera D, Wilson K, Sima J, Rivera-Mulia JC, Trevilla-García C, Nogues C, Nafie E, and Gilbert DM (2018). Genome-wide analysis of replication timing by next-generation sequencing with E/L Repli-seq. Nat. Protoc 13, 819–839. 10.1038/nprot.2017.148. 29599440 PMC6044726

[90] Kent WJ, Zweig AS, Barber G, Hinrichs AS, and Karolchik D. (2010). BigWig and BigBed: enabling browsing of large distributed datasets. Bioinformatics 26, 2204–2207. 10.1093/bioinformatics/btq351. 20639541 PMC2922891

[91] Kaya-Okur HS, Wu SJ, Codomo CA, Pledger ES, Bryson TD, Henikoff JG, Ahmad K, and Henikoff S. (2019). CUT&Tag for efficient epigenomic profiling of small samples and single cells. Nat. Commun 10, 1930. 10.1038/s41467-019-09982-5. 31036827 PMC6488672

[92] Buenrostro JD, Wu B, Litzenburger UM, Ruff D, Gonzales ML, Snyder MP, Chang HY, and Greenleaf WJ (2015). Single-cell chromatin accessibility reveals principles of regulatory variation. Nature 523, 486–490. 10.1038/nature14590. 26083756 PMC4685948

[93] Friedman J, Hastie T, and Tibshirani R. (2010). Regularization Paths for Generalized Linear Models via Coordinate Descent. J. Stat. Softw 33, 1–22. 10.18637/jss.v033.i01. 20808728 PMC2929880

