# Supplementary material for: KMT2C/KMT2D-dependent H3K4me1 mediates changes in DNA replication timing and origin activity during a cell fate transition
Source: Cell Rep. Author manuscript; Available in PMC 2025 Jun 5. (PMC12140508; doi:10.1016/j.celrep.2025.115272)
Supplement: 1 [file NIHMS2060933-supplement-1.pdf]

Cell Reports, Volume *44*

Supplemental information

KMT2C/KMT2D-dependent H3K4me1 mediates changes in DNA replication timing and origin activity during a cell fate transition

Deniz Gökbuget, Liana Goehring, Ryan M. Boileau, Kayla Lenshoek, Tony T. Huang, and Robert Blelloch

SUPPLEMENTAL FIGURES:

Fig. S1

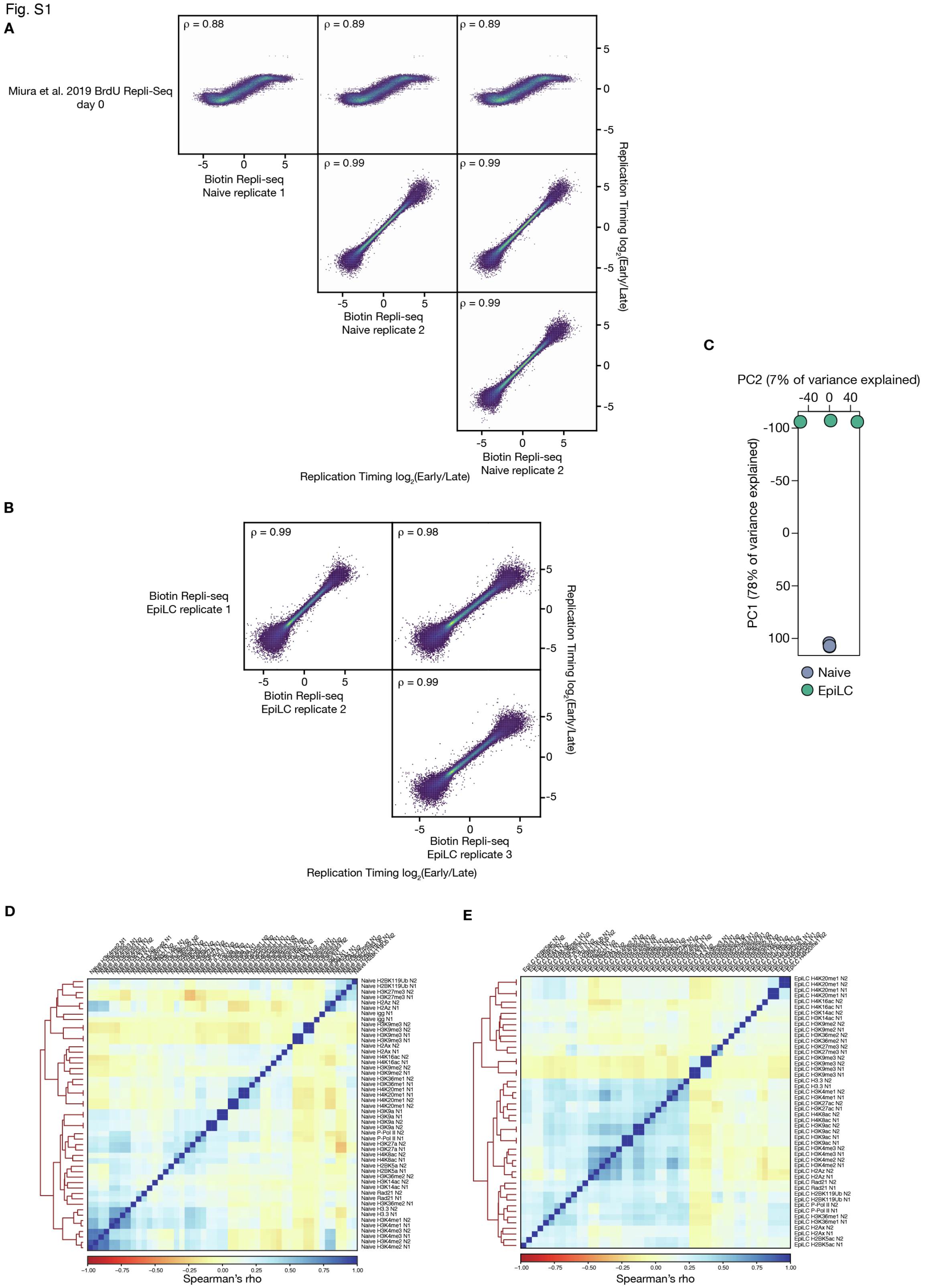

Figure S1: Quality control of BioRepli-seq and CUT&Tag. Related to Figure 1.

(A,B) Correlation plots of log_2 RT of three naïve BioRepli-seq replicates (derived from independent cultures) against each other and against previous BrdU Repli-seq method (A) (derived from equivalent culture conditions)[29] or of three EpiLC BioRepli-seq replicates (B).

(C) Principal component analysis of genome-wide naïve and EpiLC RT. First and second principal components and their variance explained shown. Replicates derived from three independent cultures shown.

(D,E) Correlation matrix of CUT&Tag data for 21 chromatin features from naïve (C) and EpiLC (D) states. Individual replicates (N1, N2) derived from independent cultures shown. Duplicate labels represent replicates split on two sequencing lanes.

Fig. S2

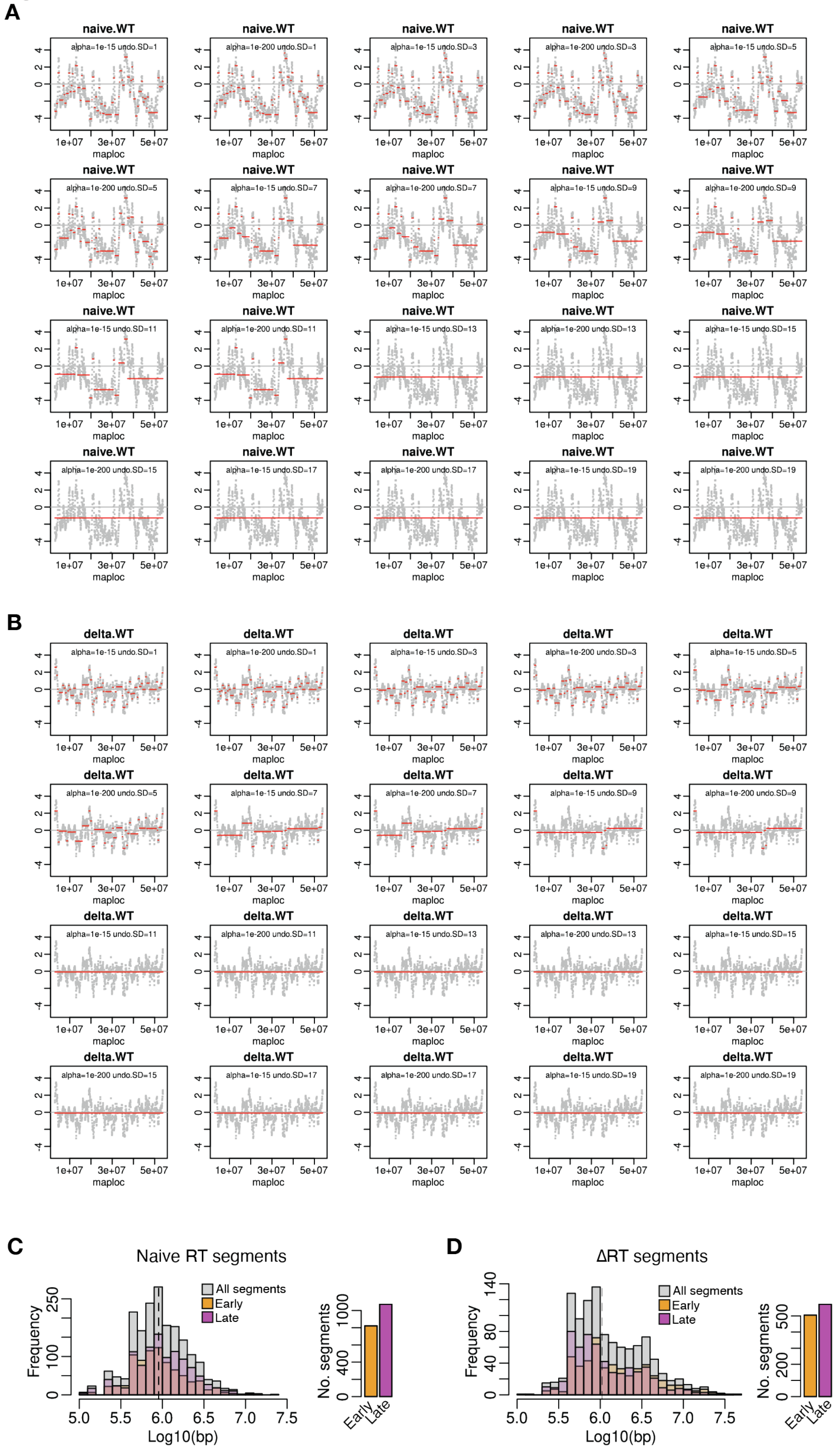
A
naive.WT
alpha=1e-15 undo.SD=1
alpha=1e-200 undo.SD=1
alpha=1e-15 undo.SD=3
alpha=1e-200 undo.SD=3
alpha=1e-15 undo.SD=5
alpha=1e-200 undo.SD=5
alpha=1e-15 undo.SD=7
alpha=1e-200 undo.SD=7
alpha=1e-15 undo.SD=9
alpha=1e-200 undo.SD=9
alpha=1e-15 undo.SD=11
alpha=1e-200 undo.SD=11
alpha=1e-15 undo.SD=13
alpha=1e-200 undo.SD=13
alpha=1e-15 undo.SD=15
alpha=1e-200 undo.SD=15
alpha=1e-15 undo.SD=17
alpha=1e-200 undo.SD=17
alpha=1e-15 undo.SD=19
alpha=1e-200 undo.SD=19
1e+07
3e+07
5e+07
maploc
B
delta.WT
C
Naive RT segments
All segments
Early
Late
Frequency
Log10(bp)
No. segments
D
ΔRT segments

Figure S2: Genome-wide RT segmentation using circular binary segmentation. Related to Figure 2.

(A,B) Parameter tuning results for 'alpha' and 'undo.SD' in circular binary segmentation algorithm for naïve (A) and ΔRT (B) segmentation shown for first one thousand 50kb bins of chr1 ("alpha = 1e-15" and "undo.SD = 5" was selected for final segmentation calls). **(C,D)** Histograms for RT segment size (left) and barplots for number of segments (right) for naïve (C) and ΔRT (D). Dashed line indicates median of all segments.

Fig. S3

A Naive RT

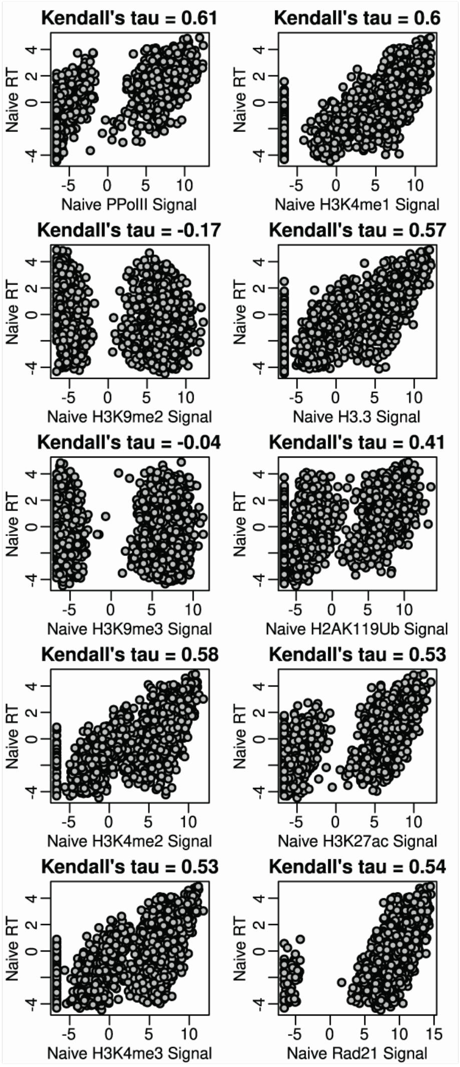

B ΔRT (EpiLC – Naive)

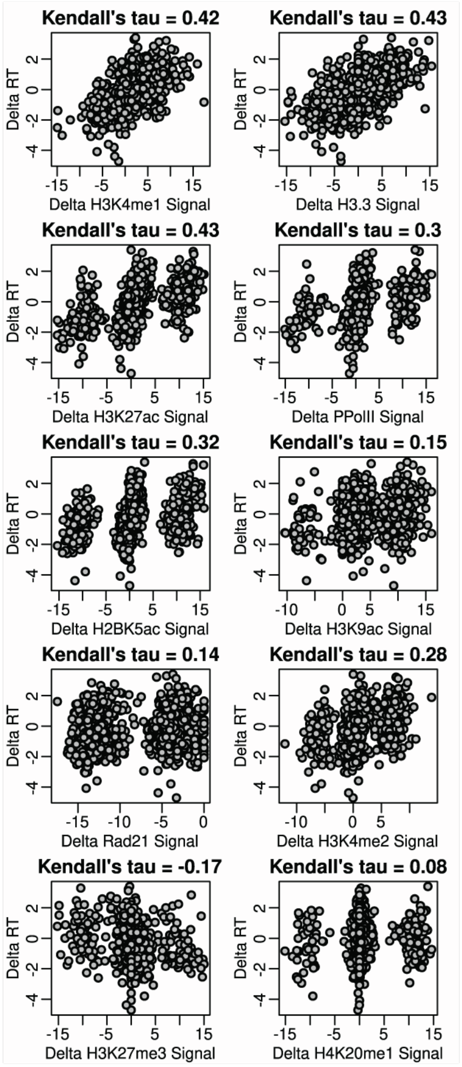

C Naive RT

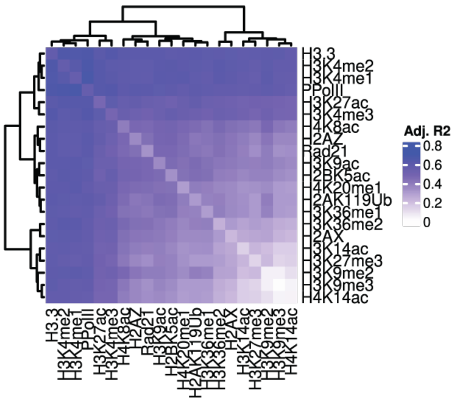

D ΔRT (EpiLC – Naive)

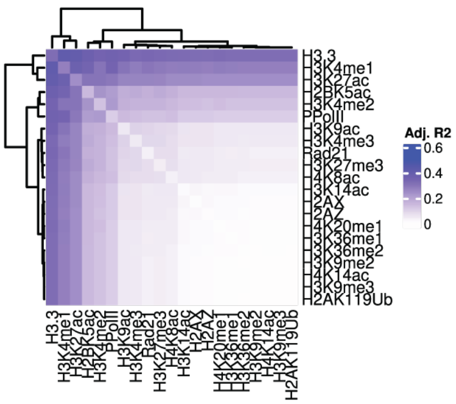

Figure S3: Associations of chromatin feature signal and RT. Related to Figure 2.

(A,B) Scatter plots of naïve or differential chromatin feature signal (Δ of EpiLC – naïve signal) versus naïve RT (A) or ΔRT segments (B), respectively, for top 10 most predictive chromatin features based on elastic net regression model parameter weights (see Figure 2D,E). Kendall's correlation coefficient shown (tau).

(C,D) Heatmap of adjusted R^2 derived from pairwise or individual (diagonal) linear regression of naïve (C) or ΔRT (D) using naïve or delta (EpiLC – naïve) chromatin feature signal, respectively.

Fig. S4

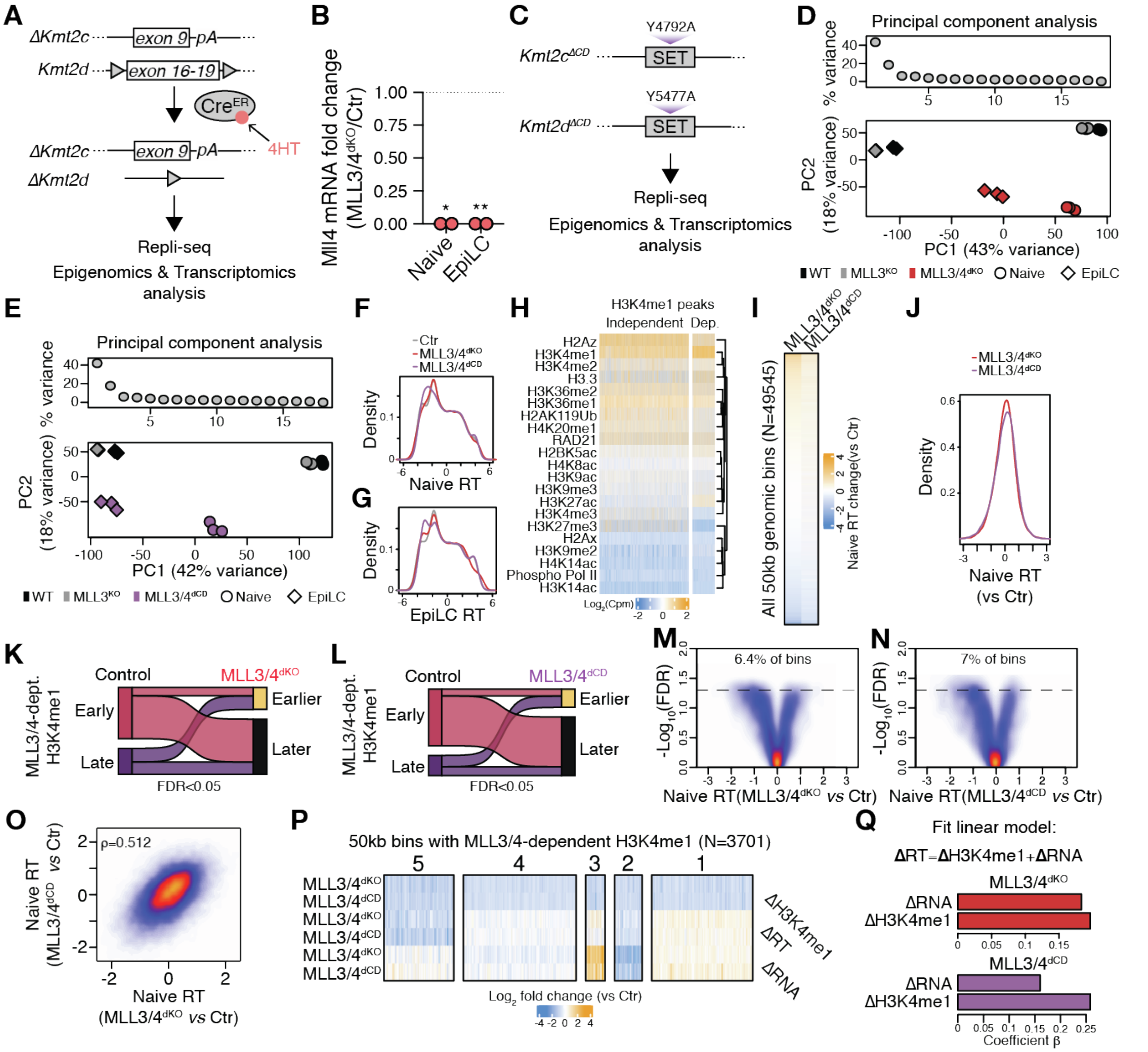

Figure S4: KMT2C/D activity locally shapes naïve RT. Related to Figure 3.

(A) Scheme of genetic model used to produce stable KMT2C/D^{dKO} ES cells.

(B) Relative Kmt2d mRNA levels in KMT2C/D^{dKO} versus KMT2C^{KO} control at naïve and EpiLC states measured by qRT-PCR. Gapdh mRNA was used as a reference.

(C) Scheme of genetic model used for stable KMT2C/D^{dCD} ES cells.

(D,E) Principal component (PC) analysis of naïve and EpiLC RT of KMT2C/D^{dKO} (D) or KMT2C/D^{dCD} (E) each compared to WT and KMT2C^{KO}. Individual replicates derived from independent cultures shown. Variance explained per PC (top) and PC1 versus PC2 shown (bottom).

(F,G) Density plots genome-wide naïve (F) and EpiLC (G) steady-state RT for controls ($KMT2C^{KO}$), $KMT2C/D^{dKO}$, and $KMT2C/D^{dCD}$.

(H) Heatmap showing clustering of the 21 analyzed chromatin features at KMT2C/D-dependent versus independent H3K4me1 peaks.

(I,J) Heatmap (J) and density plot (J) showing genome-wide RT changes for $KMT2C/D^{dKO}$ and $KMT2C/D^{dCD}$ relative to control ($KMT2C^{KO}$) in naïve state. Heatmap ordered by RT changes in $KMT2C/D^{dKO}$.

(K,L) Sankey plots for all significantly changing RT bins (*FDR* < 0.05) with KMT2C/D-dependent H3K4me1 (lost in mutants by more than 0.5 on $\log_2$ scale) for $KMT2C/D^{dKO}$ (K) or $KMT2C/D^{dKO}$ (L) compared to control in naïve state.

(M,N) Volcano plots for $\log_2$ RT fold changes versus negative $\log_{10}(FDR)$ at all genomic bins with KMT2C/D-dependent H3K4me1 (lost in mutants by more than 0.5 of $\log_2$ scale) for $KMT2C/D^{dKO}$ (M) or $KMT2C/D^{dCD}$ (N) compared to control in naïve state. Percentage of genomic bins changing shown for *FDR* < 0.05 (dashed line).

(O) Correlation plot comparing RT changes in $KMT2C/D^{dKO}$ versus $KMT2C/D^{dCD}$ relative to control in naïve ES cells. Spearman's correlation coefficient (rho) shown.

(P) Heatmap showing changes for H3K4me1, RT and transcription in $KMT2C/D^{dKO}$ and $KMT2C/D^{dCD}$ relative to control ES cells for in naïve ES cells for all 50kb RT bins that display reduction in H3K4me1 in $KMT2C/D^{dKO}$ and $KMT2C/D^{dCD}$ relative to control ES cells. Heatmap clustered by K-means.

(Q) Coefficients of linear regression explaining changes in RT in $KMT2C/D^{dKO}$ (top) or $KMT2C/D^{dCD}$ (bottom) relative to control in naïve ES cells using respective changes in H3K4me1 and transcription.

Fig. S5

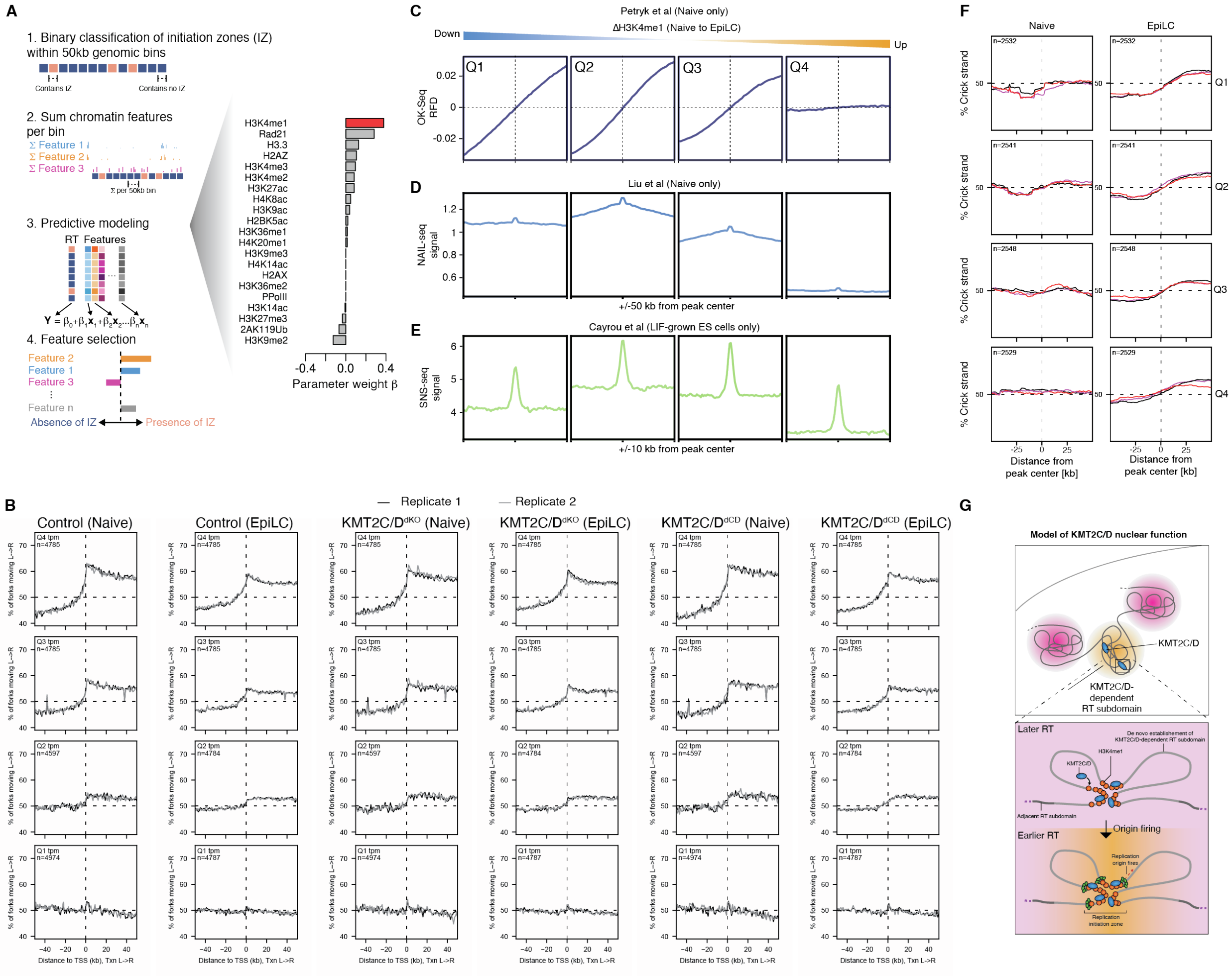

Figure S5: Predictive modeling and validation of OK-seq data. Related to Figure 4 and 5.

(A) Schematic and results for elastic net regression analysis for origin efficiency using the 21 chromatin features as predictors.

(B) Metagene analysis of OK-seq data from control (KMT2C^{KO}), KMT2C/D^{dKO}, and KMT2C/D^{dCD} cells in naïve and EpiLC states at TSSs stratified into quartiles (Q1-Q4) based on transcriptional activity. Individual replicates derived from independent cultures are shown.

(C-E) Metagene analysis of published OK-seq (C), NAIL-seq (D), and SNS-seq (E) data at H3K4me1 peaks stratified into quartiles (Q1-Q4; shown in Fig. 4C,D) based on their KMT2C/D-dependent H3K4me1 read count change in control versus mutants (average of changes in KMT2C/D^{dKO} and KMT2C/D^{dCD}) during naïve to EpiLC differentiation.

(F) Metagene analysis of OK-seq data from control (black), KMT2C/D^{dKO} (red), and KMT2C/D^{dCD} cells (magenta) at gained H3K4me1 peaks during differentiation. Peaks were

stratified into 8 quantiles (Q) based on their H3K4me1 read count change in controls during the naïve to EpiLC differentiation (see Fig. 5B for quantiles 5-8). Only gained peaks showing more than two-fold gain in H3K4me1 in controls were used as input.

(G): Model of nuclear function of KMT2C/D activity in the context of the 3D genome.
